# Copper Transport Protein Antioxidant-1 Promotes Inflammatory Neovascularization via Chaperone and Transcription Factor Function

**DOI:** 10.1038/srep14780

**Published:** 2015-10-06

**Authors:** Gin-Fu Chen, Varadarajan Sudhahar, Seock-Won Youn, Archita Das, Jaehyung Cho, Tetsuro Kamiya, Norifumi Urao, Ronald D. McKinney, Bayasgalan Surenkhuu, Takao Hamakubo, Hiroko Iwanari, Senlin Li, John W. Christman, Saran Shantikumar, Gianni D. Angelini, Costanza Emanueli, Masuko Ushio-Fukai, Tohru Fukai

**Affiliations:** 1 Departments of Medicine (Section of Cardiology) and Pharmacology, University of Illinois at Chicago, Chicago, IL; 2 Department of Pharmacology, University of Illinois at Chicago, Chicago, IL; 3 Center for Cardiovascular Research, University of Illinois at Chicago, Chicago, IL; 4 Jesse Brown Veterans Affairs Medical Center, Chicago, IL; 5 Department of Quantitative Biology and Medicine, Research Center for Advanced Science and Technology, University of Tokyo, Tokyo, Japan; 6 Department of Medicine, University of Texas Health Science Center, San Antonio, Texas; 7 Division of Pulmonary, Allergy, Critical Care and Sleep Medicine The Ohio State University Wexner Medical Center, OH; 8 Bristol Heart Institute, School of Clinical Sciences, University of Bristol, Bristol; 9 National Heart and Lung Institute, Imperial College of London, London, UK

## Abstract

Copper (Cu), an essential micronutrient, plays a fundamental role in inflammation and angiogenesis; however, its precise mechanism remains undefined. Here we uncover a novel role of Cu transport protein Antioxidant-1 (Atox1), which is originally appreciated as a Cu chaperone and recently discovered as a Cu-dependent transcription factor, in inflammatory neovascularization. Atox1 expression is upregulated in patients and mice with critical limb ischemia. Atox1-deficient mice show impaired limb perfusion recovery with reduced arteriogenesis, angiogenesis, and recruitment of inflammatory cells. *In vivo* intravital microscopy, bone marrow reconstitution, and Atox1 gene transfer in Atox1^−/−^ mice show that Atox1 in endothelial cells (ECs) is essential for neovascularization and recruitment of inflammatory cells which release VEGF and TNFα. Mechanistically, Atox1-depleted ECs demonstrate that Cu chaperone function of Atox1 mediated through Cu transporter ATP7A is required for VEGF-induced angiogenesis via activation of Cu enzyme lysyl oxidase. Moreover, Atox1 functions as a Cu-dependent transcription factor for NADPH oxidase organizer p47phox, thereby increasing ROS-NFκB-VCAM-1/ICAM-1 expression and monocyte adhesion in ECs inflamed with TNFα in an ATP7A-independent manner. These findings demonstrate a novel linkage between Atox1 and NADPH oxidase involved in inflammatory neovascularization and suggest Atox1 as a potential therapeutic target for treatment of ischemic disease.

Copper (Cu), an essential micronutrient and catalytic cofactor, plays an important role in physiological repair processes including wound healing and angiogenesis as well as various pathophysiologies including tumor growth and inflammatory diseases such as atherosclerosis^1^,^2^,^3^,^4^,^5^. Cu directly stimulates cell proliferation and migration in cultured ECs^3^. Furthermore, angiogenic lesions in cancer and atherosclerosis have higher Cu levels in cell nuclei than normal tissues^6^. *In vivo*, Cu induces neovascularization and its concentration is increased in angiogenic tissue^6^, while Cu chelators, which have been developed to treat Wilson disease (a disease of Cu toxicity), inhibit tumor growth and angiogenic responses^1^,^2^,^3^,^4^. Of importance, clinical trials for the treatment of solid tumors by Cu chelation show efficacy in disease stabilization^7^. Cu also plays an important role in inflammatory responses involved in innate and adaptive immunity^8^,^9^, which is in part via activating NFκB^10^,^11^. Cu deficiency alters intravascular adhesion of leukocytes to the activated ECs and expression of adhesion molecules, such as ICAM-1/VCAM-1^12^,^5^,^13^. Despite a critical role of Cu in angiogenesis and inflammation, the molecular mechanisms that underlie these functions are entirely unknown.

Since excess Cu is toxic, bioavailable Cu level is tightly controlled by Cu transport proteins including cytosolic Cu chaperone Antioxidant-1 (Atox1) which obtains Cu via Cu importer CTR1 and then transfers Cu to the Cu transporter ATP7A that delivers Cu to the secretory Cu enzymes for their activity, or exclude Cu^14^,^15^. Atox1 contains the conserved Cu-binding motif (MTCXXC) and Atox1-deficienct mice show reduced secretory Cu enzyme activity^16^. We previously demonstrated a novel function of Atox1 as a Cu-dependent transcription factor to regulate cell growth^17^. Indeed, Atox1 has two conserved lysines residues (Lys56 and Lys60) which are nuclear localization signal (NLS), and it is shown to be localized in the nucleus *in vitro*^17^,^18^ and *in vivo*^19^. We have shown that Atox1 binds to DNA at cis elements (GAAAGA; Atox1-responsive elements)(Atox1-RE) in the promoters and induces transactivation^17^. Cu chelator TTM, which blocks Atox1 function^20^, prevents tumor progression in clinical trials^1^,^2^,^4^.

We performed the present study to examine the role of Cu transport protein Atox1 in ischemia-induced neovascularization which is dependent on angiogenesis/arteriogenesis, inflammation, and reactive oxygen species (ROS)^21^. Here we demonstrate that Atox1 plays an essential role in inflammatory neovascularization via Cu chaperone function to increase angiogenesis via regulating extracellular matrix (ECM) modifying secretory Cu enzyme lysyl oxidase (LOX) activity as well as transcription factor function to promote ROS production and inflammatory responses via increasing NADPH oxidase organizer p47phox expression. We used Atox1^−/−^ mice with hindlimb ischemia model and sponge implant model, and various approaches including micro-CT, *in vivo* intravital microscopy, gene transfer, bone marrow transplantation (BMT), *in situ* O_2_^−^ detection, bioluminescence imaging of NFκB reporter mice, and *in vitro* reporter gene assay, and chromatin immunoprecipitation (ChIP) assay. These findings demonstrate a novel linkage between Cu transport protein Atox1 and NADPH oxidase involved in inflammatory angiogenesis and provide insight into Atox1 as a potential therapeutic target for inflammation-dependent ischemic cardiovascular diseases.

## Results

**Figure 1:**
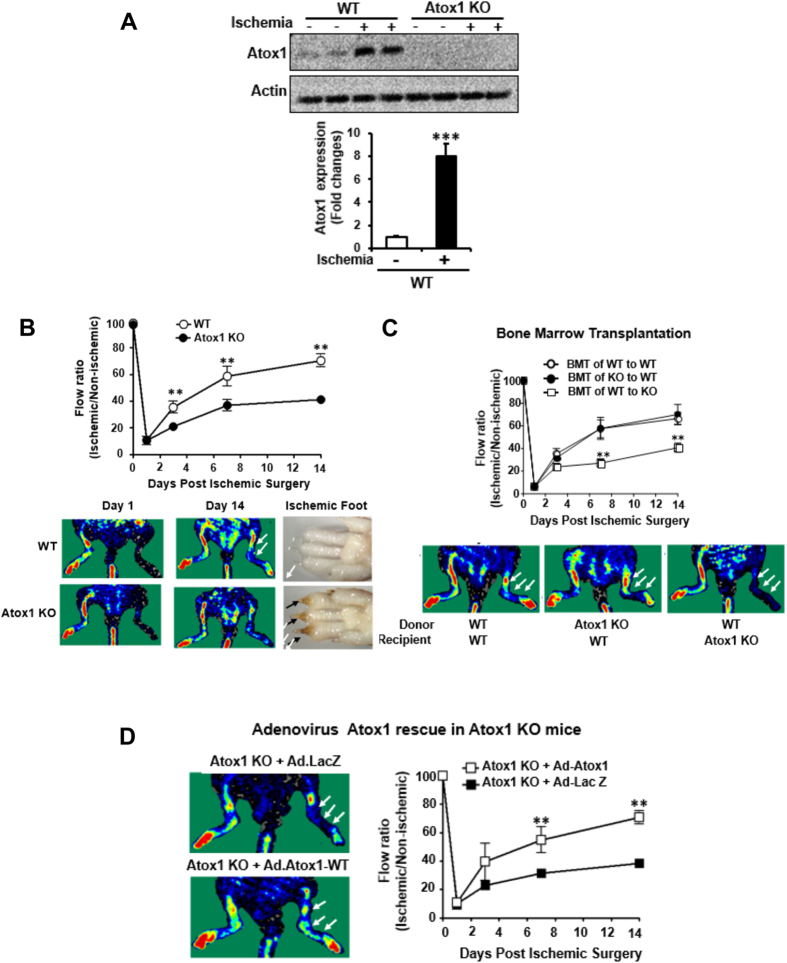
Atox1 in tissue resident cells, but not BM cells, is required for ischemia-induced neovascularization. (**A**) Atox1 protein expression in ischemic and non-ischemic muscles from wild type (WT) and Atox1 knockout (KO) mice at day 7 post-surgery. Actin is loading control (n = 5). (**B**) Blood flow recovery after hindlimb ischemia in WT and Atox1 KO mice, as determined by the ratio of foot perfusion between ischemic (left) and non-ischemic (right) legs. Bottom panels show representative laser Doppler images (white arrows shows ischemic foot) and toe necrosis characterized by edematous fingers and degenerative nail beds (black arrows) in Atox1 KO mice (n = 10). (**C**) Bone marrow transplantation (BMT) showing a role of Atox1 in tissue resident cells for ischemia-induced neovascularization. After 6 weeks of BMT, mice were subjected to hindlimb ischemia and limb blood flow was measured (n = 6). (**D**) Atox1 gene transfer in Atox1 KO mice rescues blood flow recovery after hindlimb ischemia. Purified adenoviruses (Ad.Atox1-WT, Ad. LacZ (control), 1 × 10^9^ pfu) were injected into the adductor and gastrocnemius muscles in Atox1 KO mice at one day prior to ischemic injury, and limb blood flow was measured. Representative laser Doppler images (white arrows shows ischemic foot) and quantitative analysis are shown (**C,D**). **p < 0.01, ***p < 0.001.

Atox1 in tissue resident cells is required for ischemia-induced neovascularization. To gain insight into the role of Atox1 in ischemia-induced neovascularization, we first looked at Atox1 expression in mice and patients with CLI. Atox1 protein was significantly increased after hindlimb ischemia of mice (Fig. 1A) and it was expressed in both ECs and inflammatory cells (Supplementary Fig. 1). Consistently, ischemic limb muscle samples from CLI patients showed approximately 40-fold increase in Atox1 mRNA as compared to healthy subjects (p < 0.01) (Supplementary Fig. 2) (For detailed patient characteristics see Supplementary Table 1). To examine the role of endogenous Atox1 in post-ischemic neovascularization, wild-type (WT) and Atox1^−/−^ mice were subjected to unilateral hindlimb ischemia. All Atox1^−/−^ mice survived after ischemic surgery. Figure 1B showed that the blood flow recovery after ischemia was significantly reduced in Atox1^−/−^ mice as compared to WT mice. Of note, ischemic foot showed increased toe necrosis in Atox1^−/−^ mice (Fig. 1B) with enhanced muscle damage and delayed repair at day 14 (data not shown). To examine the relative role of Atox1 in tissue resident cells and BM cells in post-ischemic neovascularization, we performed BMT. Lethally irradiated Atox1^−/−^ mice transplanted with WT-BM, but not irradiated WT mice reconstituted with Atox1^−/−^ BM, showed impaired limb perfusion recovery compared to control group (WT mice reconstituted with WT-BM) (Fig. 1C). In addition, direct injection of adenovirus expressing Atox1 (Ad.Atox1) into Atox1^−/−^ ischemic limbs significantly improved blood flow recovery compared to Atox1^−/−^ mice treated with Ad.LacZ (control) (Fig. 1D). These results suggest that Atox1 in tissue resident cells is required for ischemia-induced revascularization.

### Atox1 is required for ischemia-induced arteriogenesis and angiogenesis

**Figure 2:**
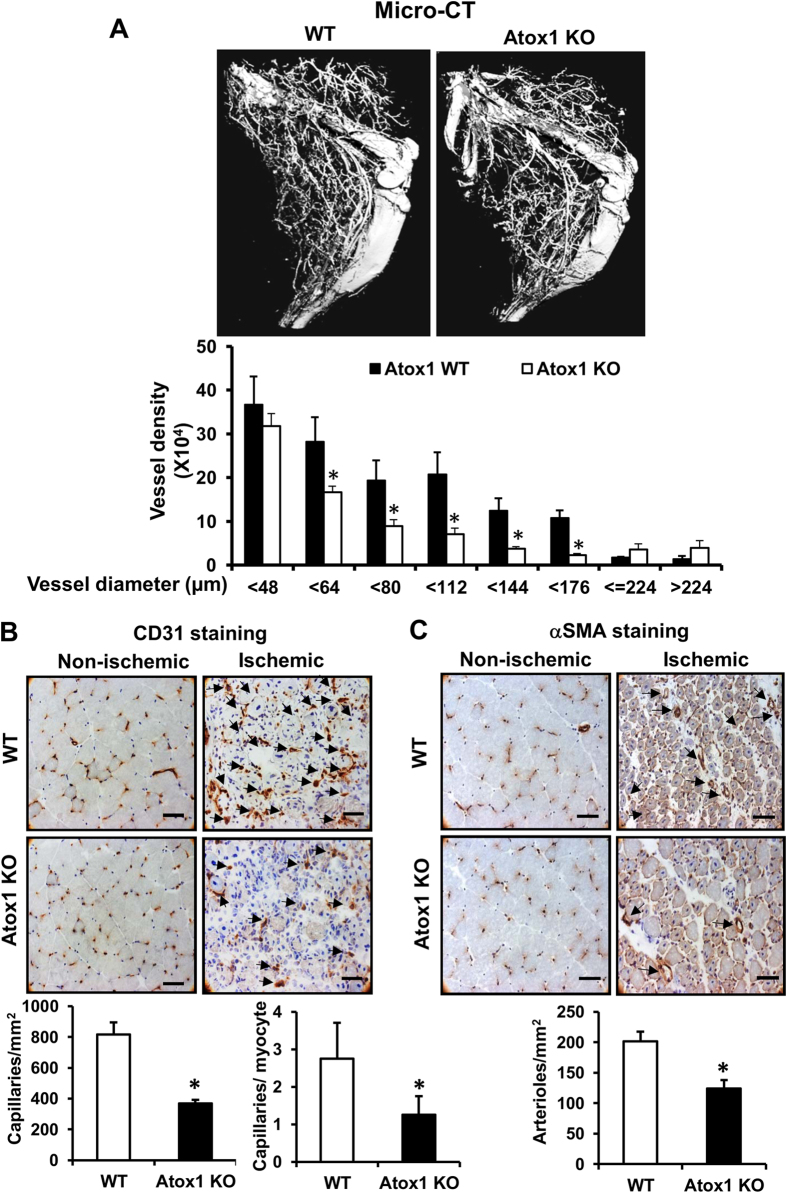
Atox1 is required for ischemia-induced arteriogenesis and angiogenesis. (**A**) Representative micro-computed tomography (micro-CT) angiograms (16 μm resolution; arteriogenesis) of ischemic legs in WT and Atox1 KO mice at day 7 post-surgery. Bottom panel shows quantitative analysis of micro-CT, as the total number of vascular structures in 1355 z-axis slices over the ranges of sizes from 4 different mice. (**B**,**C**) Representative images of CD31 (EC marker)(**B**) and αSMA (arteriole)(**C**) staining in ischemic and non-ischemic gastrocnemius muscles of WT and Atox1 KO mice at day 7. Scale bars = 50 μm. Bottom panels show their quantitative analysis (n = 4). *p < 0.05.

We next examined a role of Atox1 in arteriogenesis and angiogenesis, which contributes to ischemia-induced neovascularization. Micro-CT analysis at day 7 after ischemia showed significant decrease in the number of arterial density in Atox1^−/−^ ischemic limbs (Fig. 2A). Moreover, immunohistochemical analysis showed that the numbers of CD31^+^ECs (Fig. 2B) and αSMA^+^ arterioles (Fig. 2C) in ischemic muscles were significantly decreased in Atox1^−/−^ mice.

### Atox1 promotes angiogenesis via activating Cu enzyme LOX in ECs in an ATP7A-dependent manner

**Figure 3:**
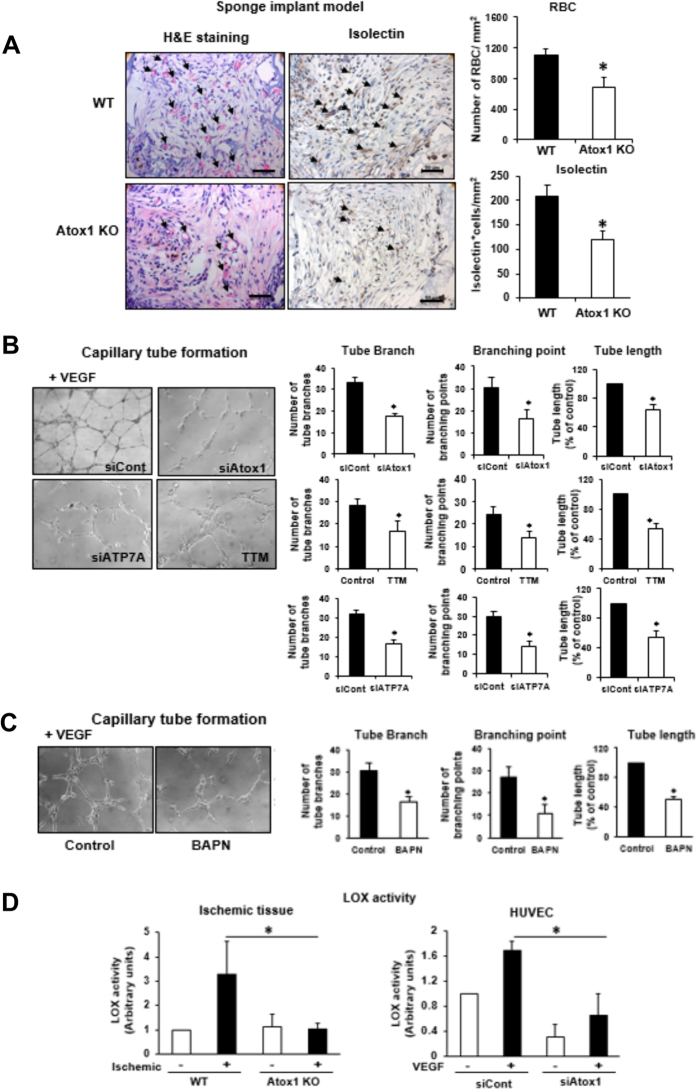
Atox1 promotes angiogenesis via activating Cu enzyme lysyl oxidase in ECs in an ATP7A-dependent manner. (**A**) Sponge implant assay was performed by implanting polyvinyl alcohol sponge containing VEGF subcutaneously into WT and Atox1 KO mice. Representative images for H&E staining and isolectin immunostaining for blood vessel formation in sponges harvested on day 21. Right panels show quantitative analysis of the number of red blood cells (RBC) and isolection+ ECs. Scale bars = 50μm. (**B**) HUVECs were transfected with control, Atox1 or ATP7A siRNAs or treated with Cu chelator TTM (20 nM, 24 hrs) and seeded on Matrigel-coated plates in culture media containing VEGF for 6 h. Four random fields per well were imaged, and representative pictures are shown (left). Averaged numbers of capillary tube branches, branching points, and tube length per field are shown (Right). (**C**) HUVECs were treated with LOX inhibitor β-aminopropionitrile (BAPN, 100 μM) for 24 hrs and capillary tube formation was measured (n = 3). (**D**) Activity of LOX was measured in ischemic gastrocnemius muscle of WT and Atox1 KO mice (left)(n = 4) or in culture medium from VEGF (20ng/ml)-stimulated HUVECs transfected with siControl or siAtox1 (right)(n = 4). *p < 0.05.

To demonstrate further a role of Atox1 in angiogenesis, we used a mouse sponge implant model^22^. Figure 3A showed that Atox1^−/−^ mice exhibited marked decrease in new blood vessel formation assessed by number of red blood cells and isolectin positive capillaries^23^. To assess the mechanism by which Atox1 is involved in angiogenesis, we first examined a role of Atox1 in cell proliferation, because we previously showed that Atox1 functions as a Cu-dependent transcription factor for cyclin D1 to regulate cell growth^17^. However, either BrdU labeling or cyclin D1 expression was not altered in Atox1^−/−^ mice (Supplementary Fig. 3). We then examined a role of Atox1 in VEGF-induced capillary-network formation, an *in vitro* model of angiogenesis. Figure 3B showed that depletion of Atox1 using siRNA in human umbilical vein endothelial cells (HUVECs) significantly reduced the number of capillary tube branches, branching sprouts as well as tube length. Similar inhibitory effects were observed by Cu chelator TTM or siRNAs for Cu importer CTR1 (Supplementary Fig. 4) or Cu transporter ATP7A.

Cu chaperone function of Atox1 is required for activating secretory Cu enzymes such as ECM-modifying Cu enzyme LOX which is mediated through Cu delivery via ATP7A. Thus, we next examined a role of LOX in capillary tube formation. Figure 3C showed that a specific LOX activity inhibitor, aminopropionitrile (BAPN), significantly reduced the number of capillary tube branches, branching sprouts as well as tube length. Moreover, LOX activity was markedly increased in ischemic tissues as well as VEGF-stimulated ECs, which were inhibited by Atox1-/- mice and Atox1 siRNA-treated HUVECs, respectively (Fig. 3D). These findings suggest that Cu chaperone function of Atox1 is involved in VEGF-induced angiogenesis via activating LOX through the CTR1-Atox1-ATP7A pathway^14^ in ECs.

### Atox1 is required for inflammatory cell recruitment to the ischemic sites *in vivo*

**Figure 4:**
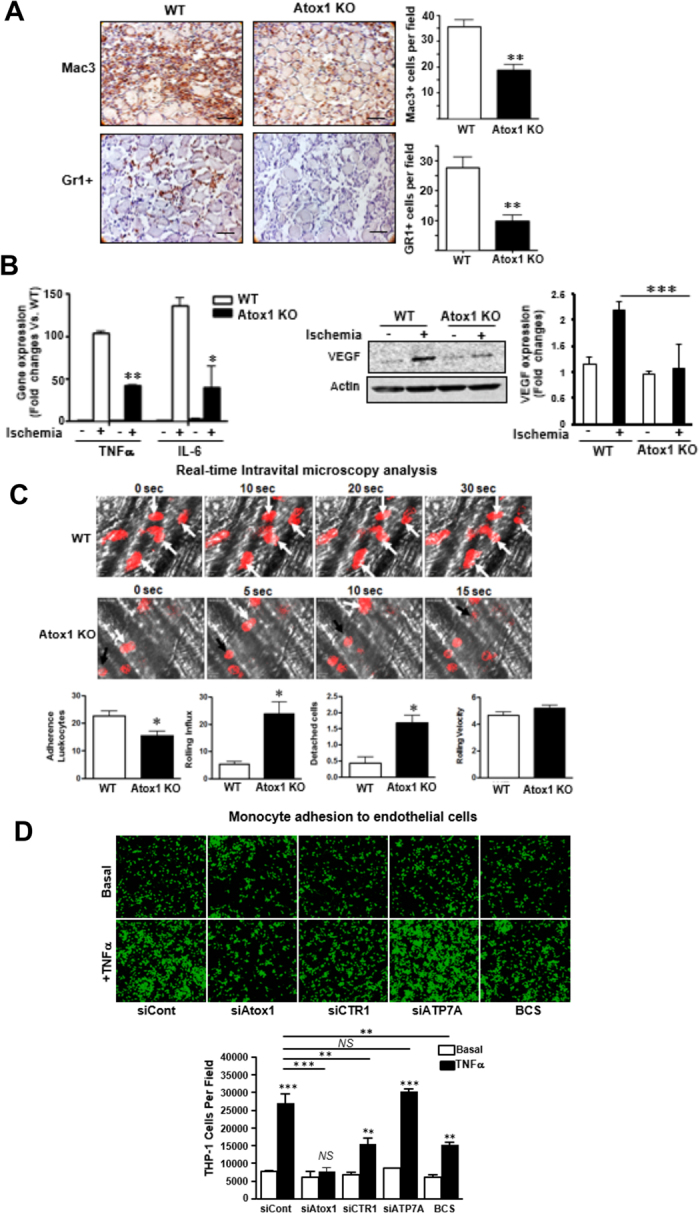
Atox1 is required for inflammatory cell recruitment to the ischemic tissues or inflamed ECs. (**A**) Representative images (left) and quantification (right) for Mac3 (for macrophage) and Gr1 (for neutrophils) staining in ischemic muscles in WT and Atox1 KO mice at day 3 (n = 4). (**B,C**) Expression of TNFα and IL-6 mRNAs (**B)**, (n = 4) and VEGF protein (**C**), (n = 3) in non-ischemic and ischemic muscles of WT and Atox1 KO mice at day 3. (**D**) Intravital microscopy analysis for neutrophil rolling and adhesion on TNFα-inflamed endothelium *in vivo*. At 3 hours after TNFα injection to WT and Atox1 KO mice, the cremaster muscle was exposed. Mouse neutrophils were monitored in the inflamed cremaster muscle venules by infusion of Alexa 647-labeled anti-Gr-1. Time lapse are shown in WT (adherent cells shown in white arrows) vs. Atox1 KO mice (the rolling cells shown in black and white arrows). In bottom, quantification of the number of adherent cells over 5 min and the number of detaching cells after firm adhesion, rolling influx (cells/min) and velocity (mm/sec) of WT and Atox1 KO neutrophils. (n = 15–16 venules in WT and Atox1 KO mice). (**E**) Confluent HUVEC monolayers transfected with Atox1, CTR1, ATP7A, or control siRNAs or treated with the Cu chelator BCS (200 μM) for 48 hrs were stimulated with TNFα (10 ng/ml) for 18 hours. The numbers of bound THP1 monocytes (fluorescently labeled) to ECs were measured with a fluorescence microscope. Bottom panel showed quantification (n = 15). *p < 0.05, **p < 0.01, ***p < 0.001

Since ischemia-induced neovascularization also depends on inflammation, we examine the role of Atox1 in inflammatory cells recruitment. Figure 4A showed that the numbers of Gr-1+ neutrophils and Mac-3+ macrophages infiltrated in the ischemic muscles were significantly decreased in Atox1^−/−^ mice. This was associated with reduced expression of inflammatory cytokines TNFα and IL-6 (Fig. 4B) as well as VEGF protein (Fig. 4C) which were mainly secreted by the infiltrated macrophages^24^. By contrast, VEGF receptor2 expression was not changed in ischemic tissue of Atox1^−/−^ mice (data not shown). Atox1 gene transfer into Atox1^−/−^ mice increased macrophages infiltration to the ischemic sites (Supplementary Fig. 5A) and reduced muscle necrosis (not shown), thereby restoring post-ischemic limb perfusion (Fig. 1D). Moreover, sponge implant model also showed that macrophage infiltration was significantly inhibited in Atox1 ^−/−^ mice (Supplementary Fig. 5B). These results suggest that Atox1 in tissue resident cells plays an important role in inflammatory cell recruitment, and thus promoting neovascularization.

Since inflammatory cell accumulation into sites of injury is dependent on leukocytes adhesion to ECs, we examined a role of Atox1 for neutrophil adhesion to the inflamed ECs *in vivo* using real-time intravital microscopy with a mouse inflammation model^25^ (Fig. 4D and Supplementary Video 1). Mouse neutrophils were monitored by infusion of the Alexa Fluor 647-labeled anti-Gr-1 antibody^26^. Neutrophil adhesion was increased in inflamed venule stimulated with TNFα, which was significantly inhibited in Atox1^−/−^ mice. By contrast, the neutrophils rolling influx over the inflamed venule wall was increased in Atox1^−/−^ mice compared to WT mice while there was no difference of the rolling velocity of neutrophils between WT and Atox1^−/−^ mice. Furthermore, the number of detaching neutrophils after firm adhesion over 30 sec increased to 3.8-fold in Atox1^−/−^ mice. These results suggest that Atox1 plays an important role in neutrophil adhesion to the inflamed endothelium, but not in neutrophil rolling *in vivo*.

### Atox1 is required for monocyte adhesion to inflamed ECs in a Cu dependent, but ATP7A-independent manner

To determine the mechanism by which Atox1 in ECs promotes inflammatory cell recruitment, we examined a role of Atox1 in monocytes adhesion to inflamed ECs *in vitro* using THP-1 monocytes and cultured HUVECs. Figure 4E showed that adhesion of THP-1 cells to HUVEC activated by inflammatory cytokine TNFα was significantly inhibited in Atox1-depleted ECs with siRNA or ECs treated with Cu chelator BCS or Cu importer CTR1 siRNA, but not by Cu transporter ATP7A siRNA. These results suggest that Atox1 is involved in monocyte adhesion to the activated ECs in a Cu-CTR1-dependent manner but independent of Cu chaperone function of Atox1 which requires ATP7A.

### Atox1 is required for adhesion molecules expression in a Cu-dependent manner via activating NFκB in inflamed ECs

**Figure 5:**
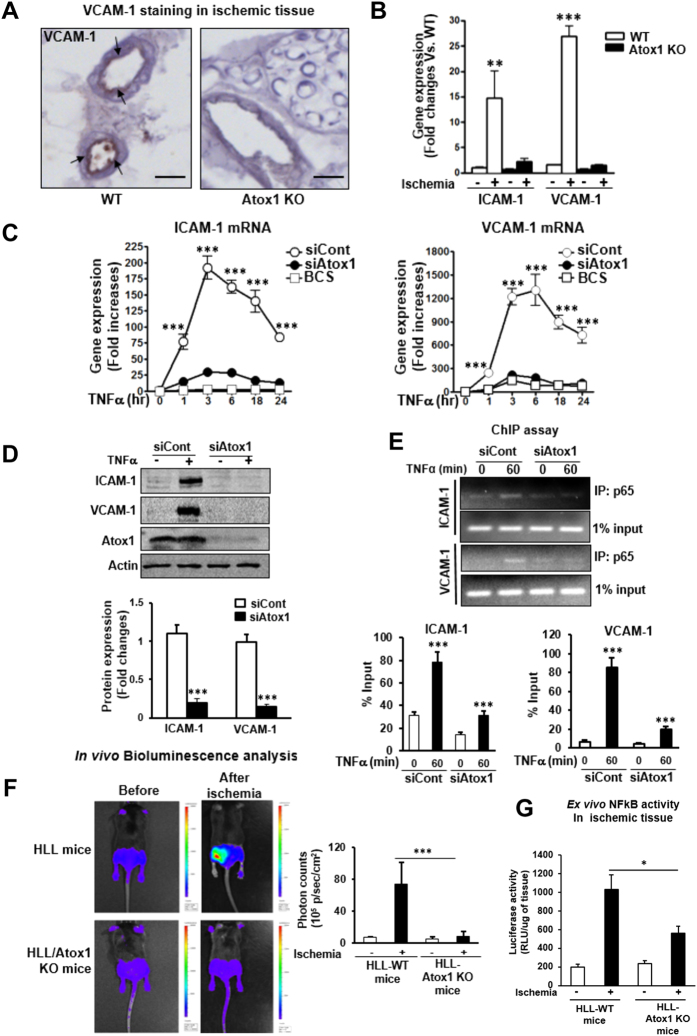
Atox1 is required for adhesion molecules expression in a Cu-dependent manner via activating NFκB in inflamed ECs. (**A**) Representative images for VCAM-1 staining in ischemic adductor muscles in WT and Atox1 KO mice at day 1 (n = 4). Scale bars = 100μm. (**B**) Expression of ICAM-1 and VCAM-1 mRNAs in ischemic and non-ischemic muscles at day 3 (n = 4). (**C**) HUVECs transfected with Atox1 or control siRNAs, or treated with BCS were stimulated with TNFα (10ng/ml), and ICAM-1 and VCAM-1 mRNAs were measured (n = 12). (**D**) HUVECs transfected with Atox1 or control siRNAs were incubated with TNFα for 18 hours, and VCAM-1 and ICAM-1 protein expression was measured. Actin was loading control (n = 8). (**E**) ChIP assay showing a role of Atox1 in TNFα-induced p65 NFkB binding to the ICAM-1/VCAM-1 promoter *in vivo*. HUVECs transfected with Atox1 or control siRNAs stimulated with TNFα were precipitated with the anti-p65NFkB antibody. The ICAM-1/VCAM-1 promoter region was amplified by PCR. Input of nuclear DNA was used as PCR control. (n = 4). (**F**) *In vivo* bioluminescence imaging of NFkB reporter mice (HLL mice) and HLL mice crossed with Atox1 KO mice before and after hindlimb ischemia at day 3. (n = 3). Representative images (left) and quantification of bioluminescence intensity (right). (**G)** Luciferase activity in homogenates of non-ischemic and ischemic gastrocunemious tissue from HLL and HLL/Atox1 KO mice at day 3 (n = 5). *p < 0.05, **p < 0.01, ***p < 0.001

Leukocyte/monocyte adhesion to the activated ECs is mediated through adhesion molecules ICAM-1 and VCAM-1^27^,^28^. Figure 5 showed that ischemia-induced expression of ICAM-1 and VCAM-1 mRNAs (Fig. 5B) and VCAM-1 protein in the endothelial layers of vessels (Fig. 5A) were abolished in Atox1^−/−^ mice. Consistent with this, in cultured ECs, increase in ICAM-1 and VCAM-1 mRNA (Fig. 5C) and protein expression (Fig. 5D and Supplementary Fig. 7A) induced by TNFα was significantly inhibited in HUVECs transfected with siRNAs for Atox1 or CTR1 or treated with Cu chelator BCS (Fig. 5C,D and Supplementary Fig. 6) or Atox1^−/−^ ECs (Supplementary Fig. 7A). These results suggest that Atox1 is involved in upregulation of ICAM-1/VCAM-1 expression in inflamed ECs, which may promote inflammatory cell recruitment to the ischemic tissue. Since transcription of ICAM-1/VCAM-1 is dependent on NFκB^27^,^28^, we next examined a role of Atox1 in NFκB activation. Atox1 siRNA did not affect TNFα-induced degradation of IκB, which is involved in activation of NFκB (data not shown). By contrast, ChIP assays showed that siAtox1 blocked NFκB subunit p65/RelA binding to the ICAM-1/VCAM-1 promoters in HUVECs stimulated by TNFα (Fig. 5E). Furthermore, *in vivo* Bioluminescence imaging of NFκB reporter mice (HLL mice) demonstrated that NF-kB activation induced by ischemia was blocked in HLL mice crossed with Atox1^−/−^ mice compared to control HLL mice (Fig. 5F). In parallel, *ex vivo* luciferase assay of ischemic and non-ischemic muscles confirmed that ischemia-induced NFκB activity was significantly inhibited in HLL-Atox1^−/−^ mice (Fig. 5G).

### Atox1 is required for endothelial ROS production in ischemic tissues *in vivo* and TNFα-induced ROS production in ECs in a Cu-dependent and ATP7A-independent manner

**Figure 6:**
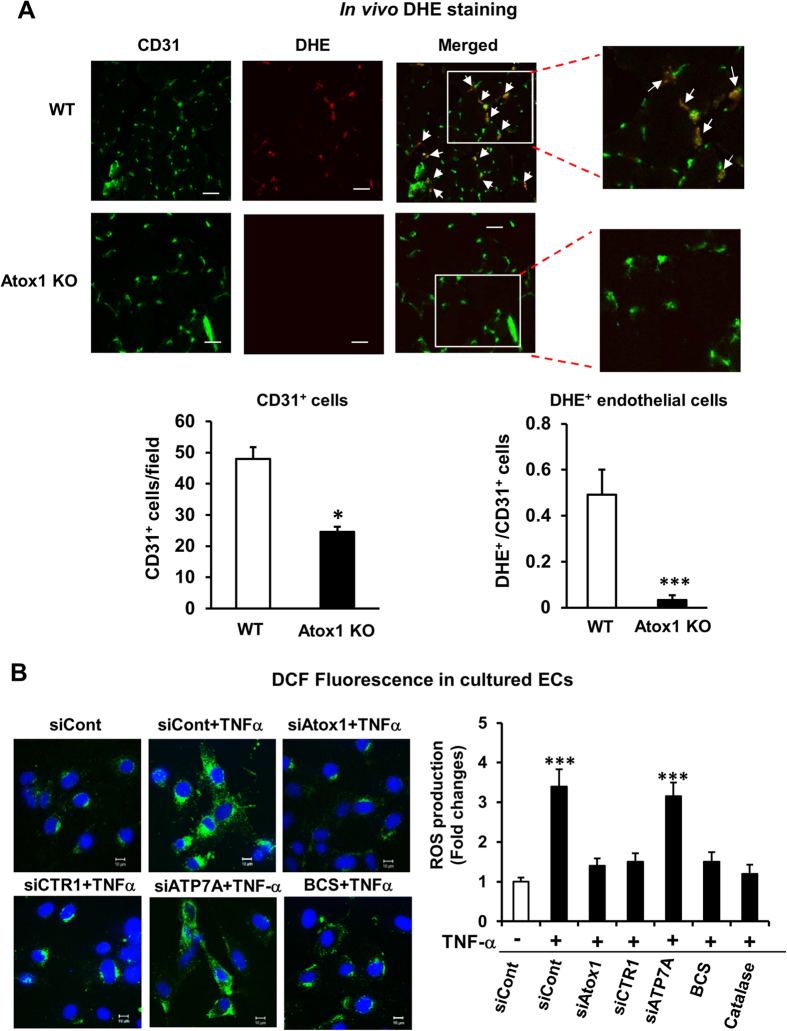
Atox1 is involved in endothelial ROS production in ischemic tissues and ECs stimulated by TNFα in a Cu-dependent and ATP7A-independent manner. (**A**) O_2_^•-^ detection probe, dihydroethidium (DHE) was injected into mice at 30 min before sacrifice at day 7, and tissues were stained with CD31. Representative pictures for CD31 staining, DHE fluorescence, and their merged images in ischemic muscles in WT and Atox 1 KO mice (10μm thickness). DHE+/CD31+ double positive cells (yellow) are shown in white arrows. Bars = 20μm. Lower panel shows quantification of number of CD31+ cells and ratio of CD31+/DHE+ cells (n = 3). (**B**) HUVECs transfected with siRNAs for Atox1, CTR1, ATP7A, control, or treated with either BCS (200uM for 48hrs) or PEG-catalase (500U/ml for 1 hr) were stimulated with TNFα (10ng/ml) for 18hrs. Representative images for DCF fluorescence and DAPI staining (blue, nucleus marker) (left) and quantification of fluorescence intensity (right) (n = 3). *p < 0.05 and ***p < 0.001.

Since ICAM-1 and VCAM-1 are redox sensitive genes and ROS are involved in NFκB activation^27^,^28^, we then examined a role of Atox1 in ROS production. Figure 6A showed that injection of O_2_^•−^ specific dye, DHE^29^ into the mice revealed that extent of decrease in DHE^+^/CD31^+^ cells (~90%) was much higher than that of CD31^+^ cells (~54%) in Atox1^−/−^ mice compared to WT mice, suggesting that Atox1 is involved in ischemia-induced endothelial ROS production. To confirm this finding *in vitro*, we next examined a role of Atox1 in H_2_O_2_ production in cultured ECs using DCF-DA which mainly detects intracellular H_2_O_2_^30^. Figure 6B showed that TNFα-induced increase in H_2_O_2_ production was blunted by siRNAs for Atox1 or CTR1, or PEG-catalase or Cu chelator BCS, but not by ATP7A siRNA. These results suggest that Atox1 is involved in ROS production in inflamed ECs in a Cu-dependent manner, but independent of Cu chaperone function through ATP7A.

### Atox1-mediated p47phox transcription is required for adhesion molecule expression in a Cu- dependent manner in ECs

**Figure 7:**
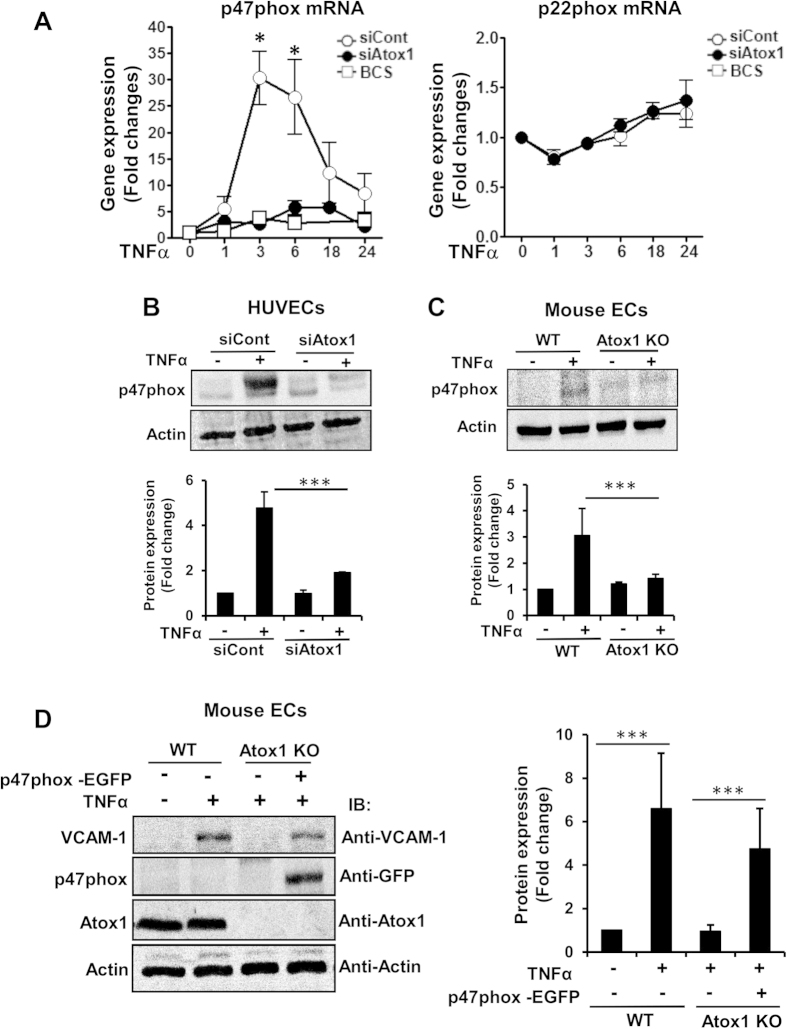
Atox1-mediated p47phox transcription is required for adhesion molecule expression in a Cu-dependent manner in ECs. (**A**) HUVECs transfected with Atox1 or control siRNAs, or treated with BCS (200uM for 48 hours) were stimulated with TNFα (10ng/ml), and p47phox and p22phox mRNAs were measured (n = 4). (**B,C**) p47phox protein expression in HUVECs transfected with Atox1 or control siRNAs (**B**) or ECs isolated from WT and Atox1 KO mice stimulated with TNFα (C)(n = 3). (**D**) ECs isolated from WT and Atox1 KO mice were transfected p47 phox-EGFP plasmid, and stimulated with TNFα for 18 hrs. Lysates were used to measure VCAM-1, p47phox-EGFP, and actin proteins by western analysis (n = 3). *p < 0.05, ***p < 0.001.

To determine the mechanism by which Atox1 is involved in ROS production in ECs, we examined a role of Atox1 in expression of NADPH oxidase, a major source of ROS involved in angiogenesis^31^. We found that TNFα-induced p47phox mRNA in HUVECs was almost completely inhibited by siRNAs for Atox1 or CTR1 or treatment with BCS (Fig. 7A and Supplementary Fig. 6) while Atox1 siRNA did not reduce p22 phox mRNA (Fig. 7A) or Nox2 or Nox4 mRNA (Supplementary Fig. 8). We confirmed that TNFα-induced p47phox protein was inhibited in Atox1 si-treated HUVECs (Fig. 7B) or Atox1^−/−^ ECs (Fig. 7C), and that ischemia-induced p47phox protein was markedly reduced in Atox1^−/−^ mice (Supplementary Fig. 7B). Of importance, inhibition of TNFα-induced VCAM-1 expression in Atox1^−/−^ ECs was rescued by re-expression of p47phox-EGFP (Fig. 7D), supporting the specificity of the Atox1-p47phox axis for increasing adhesion molecule expression.

### Atox1 functions as a Cu dependent transcription factor for p47phox in ECs

**Figure 8:**
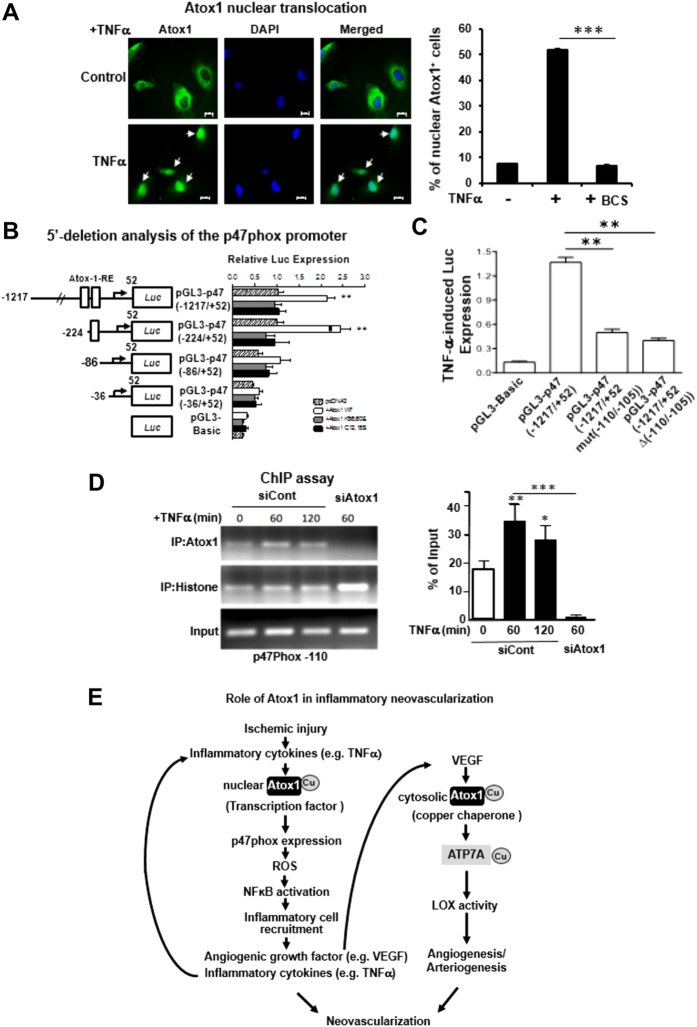
Atox1 promotes p47phox transcription by binding to its promoter in ECs activated by TNFα in a Cu-dependent manner. (**A**) Immunofluorescence staining of Atox1. HUVECs stimulated with TNFα (10 ng/ml) pretreated with or without Cu chelator BCS were immunostained for Atox1 or nuclear marker DAPI. Percentage of Atox1 + cells in nucleus was shown in right (n = 3). (**B**) Identification of Atox1 responsive elements (Atox1-RE) in a p47phox promoter. HEK293 cells were transiently transfected with indicated promoter luciferase constructs along with pcDNA/Atox1-WT or mutants Atox1 mutated at nuclear translocation signal (K56,60E) or Cu-binding domains (C12,15S). Two days after transfection, the luciferase activity was measured (n = 3). (**C**) HUVECs were transfected with a p47phox promoter luciferase reporter construct (pGL3-p47phox promoter (−1217/+52)) with or without mutation or deletion of the Atox1-RE (−110 to −105 region) (n = 3). (**D**) ChIP assay showing Atox1 binding to the p47phox promoter *in vivo*. HUVECs transfected with Atox1 or control siRNAs were treated with TNFα (10ng/ml). After precipitation with anti-Atox1 antibody, the p47phox promoter region (the Atox1-RE (−110 to −105 region)) was amplified by PCR (n = 4). Input nuclear DNA was used as PCR control. *p < 0.05. ^#^p < 0.05, **p < 0.01, ***p < 0.001. (**E**) Models for role of Atox1 in inflammatory neovascularization, which is dependent on arteriogenesis/angiogenesis and inflammation. Atox1 functions as a Cu chaperone mediated through ATP7A to increase LOX activity involved in VEGF-induced angiogenesis as well as a Cu-dependent transcription factor for NADPH oxidase organizer p47phox to increase the ROS-NFkB-VCAM-1/ICAM-1 axis in ECs inflamed with TNFα. This in turn promotes recruitment of inflammatory cells which secrete TNFα and VEGF. This represents a novel positive feedback loops whereby Cu transport protein Atox1 promotes inflammatory neovascularization.

Since Atox1 was involved in ROS production and monocyte adhesion to inflamed ECs in a Cu-dependent but Cu chaperone-independent manner (Figs 4 and 6), we examined whether Cu-dependent transcription factor function of Atox1 is involved in p47phox transcription in ECs. Immunofluorescence analysis revealed that Atox1 was localized in the cytosol in basal state and TNFα stimulation promoted Atox1 translocation into the nucleus (Fig. 8A), which was blocked by siCTR1 or BCS (Fig. 8A and Supplementary Fig. 9). Since p47phox promoter contains several Atox1-REs (GAAAGA)^17^,^32^ at −305, and −110 bp upstream of the transcription start site, we performed luciferase reporter assays using p47phox promoters. Figure 8B showed that p47phox promoter activity was significantly increased by overexpression of WT-Atox1, but not by Atox1 mutated at Cu-binding sites (C12, C15S) or nuclear localization signal motif (K56, 60E). A 5′-deletion analysis of the p47phox promoter identified functional Atox1-REs from −110 to −105 bp at the promoter, which had no response to the Atox1 mutants (Fig. 8B). Furthermore, mutation or deletion of the GAAAGA sequence (−110/−105) in the p47phox promoter abolished TNFα -induced activation of p47phox promoter (Fig. 8C). To test whether endogenous Atox1 binds to the p47phox promoter *in vivo*, we performed ChIP assays using specific Atox1 antibody in HUVECs (Fig. 8D). Atox1 associated with the p47phox promoter including Atox1-RE at −110 in a time-dependent manner in ECs stimulated by TNFα, which was abolished by siAtox1. These results suggest that Atox1 binds to the p47phox promoter at Atox1-RE at −110 in response to TNFα, thereby promoting H_2_O_2_ production that stabilizes p65/RelA binding to ICAM-1/VCAM-1 promoters, which in turn increases adhesion molecule expression and leukocytes/monocytes adhesion in ECs.

## Discussion

Role of Cu in angiogenesis and inflammatory responses has been appreciated^1^,^2^,^3^,^8^,^9^; however, its molecular mechanisms are entirely unknown. Here we demonstrate that Cu transport protein Atox1 plays an essential role in inflammatory neovascularization via Cu chaperone function through Cu transporter ATP7A to increase angiogenesis via regulating secretory Cu enzyme LOX mediated activity as well as Cu-dependent transcription factor function to promote ROS production and inflammatory responses via increasing p47phox expression in an ATP7A-independent manner. The following key observations support this conclusion. First, we observed that Atox1 expression is significantly upregulated in patients and mice with CLI. Second, mice lacking Atox1 exhibit impaired ischemia-induced perfusion recovery, arteriogenesis/angiogenesis and infiltration of inflammatory cells that produce angiogenic cytokines including VEGF and TNFα. Third, BMT, Atox1 gene transfer, *in vivo* intravital microscopy experiments reveal that Atox1 in ECs, but not BM cells, is required for post-ischemic revascularization and recruitment of inflammatory cells. Fourth, Atox1 functions as a Cu chaperone to promote VEGF-induced angiogenesis in ECs via increasing LOX activity in a Cu/ATP7A-dependent manner. Fifth, Atox1 translocates to nucleus in response to TNFα, resulting in binding to p47phox promoter for its transactivation, thereby promoting the ROS/NFkB-dependent ICAM-1/VCAM-1 expression in ECs in an ATP7A-independent manner, which facilitates recruitment of inflammatory cells. Thus, our study demonstrates a novel linkage between Atox1 and NADPH oxidase as well as positive feed-back loops that promote inflammatory neovascularization organized by a Cu chaperone and transcription factor function of Atox1 (Fig. 8E).

In this study, we show that Atox1 expression is significantly upregulated in mice and patients with CLI, supporting the clinical significance of Atox1 in peripheral vascular disease. Ischemia hindlimb model with micro-CT analysis and sponge implant assay reveal that Atox1 is involved in ischemia/inflammation-induced angiogenesis and arteriogenesis. We previously reported that Atox1 functions as a Cu-dependent transcription factor to stimulate cell growth via regulating cyclin D1 in fibroblast in an ATP7A-independent manner^17^. However, BrdU staining and cyclin D1 expression in ischemic tissues are not altered in Atox1^−/−^ mice. The discrepancy between our previous and current study may be attributable to differences in cell types or experimental models. We thus examined if Cu chaperone function of Atox1 which depends on Cu transporter ATP7A that delivers Cu to the secretory Cu enzymes^14^,^15^, is involved in angiogenesis. We found that activity of Cu enzyme LOX, which is involved in angiogenesis^33^,^34^, is markedly decreased in ischemic tissue of Atox1^−/−^ mice or Atox1-depleted HUVECs. VEGF-induced capillary tube formation in ECs is blocked by Cu chelator or siRNAs for CTR1 or Atox1 or ATP7A; or specific LOX inhibitor. Furthermore, we previously reported that Atox1^35^ or CTR1-ATP7A-LOX axis^35^,^36^ are involved in PDGF-induced vascular smooth muscle migration, which may contribute to arteriogenesis and collateral formation^37^. Thus, these results suggest that Atox1 plays an important role in VEGF-induced angiogenesis in ECs and post-ischemic angiogenesis/arteriogenesis at least via Cu chaperone function to increase LOX activity through the CTR1-Atox1-ATP7A pathway.

Cu deficiency is shown to inhibit vascular adhesion of leukocytes to the activated ECs^12^ and pathological angiogenesis^1^,^2^,^3^,^4^ but its mechanism remains unclear. Infiltrated inflammatory cells after ischemia are the major source of angiogenesis growth factors and cytokines, thereby promoting revascularization^24^. Here we show that ischemia-induced macrophage and neutrophil infiltration is inhibited in Atox1^−/−^ mice, which is associated with decreased expression of TNFα and VEGF. Experiments with BM transplantation and Atox1 gene transfer to Atox1^−/−^ mice of ischemic muscles reveal that Atox1 in tissue resident cells such as ECs is important for restoring limb perfusion and macrophage recruitment. This conclusion is further supported by the *in vivo* intravital microscopy study showing that exogenous labeled Atox1^+^ neutrophil adhesion to the TNFα-inflamed endothelium is inhibited in Atox1^−/−^ mice. Moreover, we previously reported that BM-derived macrophage migration is not inhibited in Atox1^−/−^ mice^35^. Thus, our data are consistent with the conclusion that Atox1 in ECs, but not BM cells, is required for recruitment of inflammatory cells that secrete TNFα and VEGF, thereby enhancing neovascularization and perfusion recovery in ischemic limbs.

Previous reports suggest that Cu plays an important role in activation of redox-sensitive transcription factor NFκB and induction of its downstream genes including adhesion molecules ICAM-1/VCAM-1 which mediate inflammatory cell adhesion to ECs^10^,^38^,^39^,^5^,^13^. However, its underlying mechanism is entirely unknown. Here we provide the first evidence that Atox1 is involved in ROS production, NFκB-DNA binding, VCAM-1/ICAM-1 expression and monocyte adhesion to ECs stimulated by TNFα in a Cu/CTR1-dependent manner. Of note, these Atox1-mediated inflammatory responses are independent of ATP7A, suggesting that Cu chaperone function of Atox1 *is not* involved. Role of Atox1 in promoting ROS and NFκB activity is further confirmed by *in vivo* studies demonstrating a significant decrease in DHE^+^/CD31^+^ ECs in ischemic tissues as well as NFκB activity measured by bioluminescence imaging of NFκB-reporter mice. Consistent with our result, we previously used EC-specific catalase transgenic mice to demonstrate that EC-derived H_2_O_2_ plays an essential role in ischemia-induced arteriogenesis/angiogenesis, NFκB-dependent adhesion molecule expression and inflammatory cells recruitment^40^. By contrast, Tirziu *et al.*^41^ reported that EC-specific overexpression of inhibitor of NFκB in mice show reduced limb perfusion and adhesion molecule expression and monocyte influx in ischemic tissues with excess branched arterial network and immature vessels. The discrepancy regarding arteriogenesis might be due to the difference between overexpression of IkBα mutant and endogenous ROS inhibition, or there are possible additional downstream targets of Atox1 than p47phox NAPDH oxidase, as discussed below.

We previously reported that Atox1 functions not only as a Cu chaperone but also as a Cu-dependent transcription factor by binding to DNA at Atox1-RE^17^,^32^. The present study demonstrates that Atox1 functions as a Cu-dependent transcription factor for p47phox, a cytosolic organizer of NADPH oxidase in ECs activated by TNFα, thereby increasing ROS production and redox-sensitive NFκB-dependent ICAM-1/VCAM-1 expression, which promotes monocytes adhesions to ECs. It is shown that p47phox is involved in adhesion molecule expression and inflammatory cell adhesion in ECs^27^,^42^,^43^,^44^ and that NADPH oxidase plays an important role in post-ischemic neovascularization in context- and disease state-specific manner^23^,^45^,^46^,^47^,^48^. In this study using mmunofluorescence, luciferase, and ChIP assays, we found that Atox1 translocates to nucleus in response to TNFα, and then bind to the Atox1-RE 17,32 in the p47phox promoter, in a Cu/CTR1-dependent manner. Moreover, overexpression of Atox1, but not Atox1 mutants lacking the Cu binding or nuclear translocation sequence, increases p47phox promoter activity. p47phox protein expression is significantly decreased in Atox1^−/−^ ECs or Atox1 siRNA-treated HUVECs stimulated by TNFα as well as ischemic tissues of Atox1^−/−^ mice. Among NADPH oxidase components, p47phox seems to be the specific target of Atox1, since expression of other subunits such as p22phox and Nox2 as well as Nox4 are not affected by siAtox1 in TNFa-stimulated ECs, and the promoter region of p47phox, but not other components of NADPH oxidase such as p22phox or Nox2, contains Atox1-RE regions at −305, and −110 bp upstream of the transcription start site. Importantly, decrease in TNFα-induced VCAM-1 expression in Atox1^−/−^ ECs is rescued by re-expression of p47phox, supporting the specificity of the Atox1-p47phox-VCAM-1 axis.

It is shown that VEGF-induced ROS play an important role in angiogenesis in ECs^31^, and that VEGF stimulation increases the expression of ICAM-1/VCAM-1^49^. However, we found that Atox1 translocation to nucleus is not observed by VEGF or other growth factors such as FGF or HGF, and that Atox1siRNA has no effects on VEGF-induced ROS production or ICAM-1/VCAM-1 expression in ECs (Supplementary Figs 10–12). As discussed above, Atox1 is involved in recruitment of inflammatory cells that produce VEGF in ischemic tissues (Fig. 4C). Thus, it is conceivable that Atox1 may be upstream of VEGF signaling linking to ROS-dependent induction of ICAM-1/VCAM-1 during ischemia-induced neovascularization, which may explain why expression of these adhesion molecules is completely suppressed in Atox1^−/−^ ischemic tissues (Fig. 8E). Moreover, in ECs stimulated with VEGF, Atox1 functions as a cytosolic Cu chaperone for LOX through ATP7A, instead of Cu-dependent transcription factor, to promote angiogenesis (Fig. 8E). Addressing molecular mechanism by which Atox1 translocates to the nucleus in response to TNFα specifically or other inflammatory cytokines is the subject of future study.

p47phox transcription is reported to be negatively regulated by binding of HBP1 to the p47phox promoter at −1300bp^50^, while it is positively regulated by binding of Ets family protein PU.1 to the p47phox promoter at −40bp^51^. Thus, these transcription factors may also participate in regulating Atox1-mediated p47phox transcription. In addition, we previously reported that Atox1 acts as a Cu-dependent transcription factor for secretory copper enzymes extracellular SOD (ecSOD) in vascular smooth muscle^19^,^32^, which plays a role in ischemia-induced reparative neovascularization by regulating extracellular ROS levels^30^,^52^. HIF1α is also Cu-dependent^53^. It is thus conceivable that Cu-dependent transcription factor Atox1 may have additional downstream targets to promote neovascularization. Atox1 functions as a Cu chaperone for ecSOD^32^ to increase its activity via binding to ATP7A^14^. However, since ecSOD is not/little expressed in cultured ECs^54^, ecSOD may not be involved in Atox1-mediated angiogenesis and inflammatory responses in ECs. Moreover, Cu-dependent angiogenesis may be mediated through increased binding of growth factors to cell surface or secretion of proangiogenic peptides such as FGF1 and IL-1α, or stabilization of extracellular matrix such as fibronectin, which are shown to be regulated by Cu^1^,^2^,^3^.

In summary, our study will enrich our understanding about the molecular mechanisms of how Cu is involved in inflammation and angiogenesis-dependent diseases. We provide the novel evidence that Atox1 plays an essential role in inflammatory neovascularization via Cu chaperone function for LOX to increase angiogenesis as well as Cu transcription factor function to promote recruitment of inflammatory cells which secrete TNFα and VEGF via the p47phox/ROS-NFkB-ICAM-1/VCAM-1 pathway. Clinical and animal studies indicate that Cu deficiency therapies by either Cu deficient diet or Cu chelation can be effective for several pathological conditions such as tumor progression and inflammatory diseases^1^. Function of ROS is dependent on its concentration; optimal levels of ROS are required, but excess ROS produced under pathological states are inhibitory for neovascularization^21^,^31^. Thus, understanding the Cu chaperone/transcription factor function of Atox1 in physiological and disease conditions will lead to new therapeutic strategy for inflammation-dependent ischemic cardiovascular diseases.

## Methods

### Animals

Atox1^−/−^ mice (backcrossed eight times to C57Bl/6J (Jackson laboratory) were obtained from Mutant Mouse Regional Resource Centers. Atox1^−/−^ mice were originally reported to have various phenotypes from perinatal death (45% of pups) to the phenotype which could survive more than one month^16^. In our laboratory, they were further backcrossed to C57Bl/6J mice more than ten times, and thus C57Bl/6J mice were used as control. “Survivor” mice were intercrossed with more than 10 times and Atox1^−/−^ mice used in the present study survived more than six months (90%). For the experiments, Atox1^−/−^ mice were weaned at 4 weeks of age and maintained on regular chow for 2 to 3 months. Diet and water were provided *ad libitum*. All studies were carried out in accordance with the guidelines approved by the Animal Care and Use Committee of the University of Illinois-Chicago

### Mouse hindlimb ischemia model

Mouse hindlimb ischemia was induced as described previously^29^,^55^. Briefly, following anesthesia (87 mg/kg ketamine, 13 mg/kg xylazine), the left femoral artery was exposed under a dissection microscope. The proximal portion of the femoral artery and the distal portion of the saphenous artery were ligated. All branches between these 2 sites were ligated or cauterized, and arteriotomy was performed. We measured ischemic (left)/nonischemic (right) limb blood flow ratio using a laser Doppler blood flow (LDBF) analyzer (PeriScan PIM 3 System; Perimed) as we described^29^,^55^. Mice were anesthetized and placed on a heating plate at 37 °C for 10 minutes to minimize temperature variation. Before and after surgery, LDBF analysis was performed in the plantar sole. Blood flow was displayed as changes in the laser frequency, represented by different color pixels, and mean LDBF values were expressed as the ratio of ischemic to nonischemic LDBF. Tissue was extracted in radioimmunoprecipitation assay lysis buffer supplemented with protease inhibitor. The lysates were clarified by ultracentrifugation (13200 rpm, 10 minutes) at 4 °C and protein were measured by bicinchoninic acid assay. For adenovirus experiment, recombinant purified adenovirus (Ad.Atox1-WT, Ad. LacZ (control), 1 × 10^9^ pfu) was injected into the adductor and gastrocnemius muscles in Atox1 KO mice at one day prior to hindlimb ischemia.

### Bone marrow (BM) transplantation

BM transplantation was performed as we previously described^29^. BM cells were isolated by density gradient separation. Recipient mice were lethally irradiated with 9.5 Gy and received an intravenous injection of 3 million donor BMCs 24 hours after irradiation. To determine the transplantation efficiency, transplantation was performed between CD45.1 mice (Jackson Laboratories) and either Atox1^−/−^ or WT mice and peripheral white blood cells were stained with anti-CD45.1 (BD) and anti-CD45.2 (BD). Hindlimb ischemia was induced 6 to 8 weeks after bone marrow transplantation.

### Histology and immunohistochemistry

Mice were sacrificed at 3–14 days after surgery, and muscles of the lower limbs or upper limbs were harvested, methanol fixed, and paraffin embedded. Frozen sections were prepared by overnight 4% PFA incubation followed by sucrose dehydration and OCT embedding. Capillary density in the ischemic muscles was determined in methanol fixed 5 μm thick sections that were stained with anti-mouse CD31 antibody (BD Biosciences) followed by biotinylated anti-mouse IgG antibody (Vector Laboratories). Arterioles were stained with anti-αSMA antibody (Sigma). Adhesion molecule expression was stained by goat anti-VCAM-1 (Santa Cruz). Monocytes/macrophages were labeled with anti-Mac3 and neutrophils with anti-Gr1 antibody followed by biotinylated anti-rat IgG (Vector Laboratories). Rabbit polyclonal anti-Atox1^17^ and rat anti-CD45 (BD Biosciences) staining was performed in frozen sections. For immunohistochemistry, we used R.T.U. Vectorstain Elite (Vector Laboratories) followed by DAB (Vector Laboratories). Images were captured by Axio scope microscope or confocal microscopy and processed by AxioVision 4.8 or Zeiss LSM510 software, respectively.

To quantify proliferating cells, mice were injected intraperitoneally with the thymidine analog 5-bromo-2′-deoxyuridine (BrdU; 40 mg/kg body weight, 500 μl, SigmaAldrich) at 12 hours and 1 hour before sacrifice asdescribed previously^52^. Mice were euthanized and perfused through the left ventricle with saline, and hindlimb muscles were embedded in OCT compound and snap-frozen in liquid nitrogen. Sections were incubated with a rat anti-BrdU antibody (Abcam) to detect proliferating cells. For nuclear staining, BrdU-labeled tissue sections were counterstained with 4′, 6-diamidino-2-phenylindole (DAPI).

### Volumetric Micro-CT

Mice were euthanized and vasculature was flushed with 0.9% normal saline containing, papaverine (4 mg/L), and adenosine (1 g/L) for 3 minutes. After vasodilation, 2% paraformaldehyde was perfused for 5 minutes at 100 mm Hg pressure to fix the vasculature and wash with saline. Contrast agent containing bismuth (0.5 mL) (Generously provided by Dr. Michael Simons, Section of Cardiovascular Medicine, Yale School of Medicine, New Haven, CT)^41^ in 5% gelatin was injected over a period of 2 minutes with a syringe pump. The mice were then immediately chilled on ice and immersion-fixed in 2% paraformaldehyde overnight. The hindlimb vasculature was imaged with a high-resolution micro-computed tomography (micro-CT) imaging system (Scanco μCT 50, Bassersdorf, Switzerland) in MicroCT/Histology Core, Rush University, Chicago, IL, USA. The samples were scanned at 70 kV voltage and 114 μA current, with a500ms integration time and 16 μm resolution. A region of interest was reconstructed using 1355 z-sections (~21.68 mm) of the hindlimb. During postprocessing of the image, a 530 gray-scale value was set as a threshold to eliminate bone with minimal sacrifice of vessel visualization. Quantification was performed by using Scanco software.

### Intravital microscopy analysis

WT (C57BL/6J) or Atox1^−/−^ mice were prepared for intravital microscopy as described previously^25^,^26^. Mice were pre-anesthetized by intraperitoneal injection of ketamine (87 mg/kg, Bedford Laboratories, Bedford, OH) and xylazine (13 mg/kg, Akorn Inc., Decatur, IL). A tracheal tube was inserted and the mouse was placed on a thermo-controlled blanket (37 °C). To maintain anesthesia, the ketamine and xylazine solution, 50 μl, was administered every 30 minute through a cannulus placed in the jugular vein. Mice were kept on a 37 °C thermo-controlled rodent blanket (Thermal Care, Niles, IL) throughout the experiment. For TNFα stimulation, mice were treated with intrascrotal injection of murine TNFα (0.5 μg in 250 μl saline) for 3 hr before exteriorization of the cremaster muscle. After the scrotum was incised, the cremaster muscle was exteriorized onto an intravital microscopy tray. The cremaster preparation was superfused with thermo-controlled (37 °C) and aerated (5% CO_2_, 95% N_2_) bicarbonate buffered saline throughout the experiment.

Three hours after TNFα injection, the cremaster muscle was exposed as described previously^25^,^26^. Mouse neutrophils were monitored in an area of 0.02 mm^2^ over 5 min in the inflamed cremaster muscle venules by infusion of an Alexa Fluor 647-labeled anti-Gr-1 antibody (0.04 μg/g body weight, BioLegend). Five to seven different venules were monitored in a mouse. Rolling and adhesion of neutrophils in post-capillary venules was recorded using an Olympus BX61W microscope with a 60x objective. Digital images were acquired using a high-speed digital camera (Hamamatsu C9300) through an intensifier (Video Scope International, Dulles, VA). Fluorescence images were analyzed using Slidebook v5.0 (Intelligent Imaging Innovations, Denvor, CO). Fluorescence images were captured at exposure times of 100 millisec and bright field images were captured with exposure times of 20 millisec.

Wall shear rate (Wsr) in the mouse cremaster muscle venule averaged 500–700 s^−1^ and was calculated from the following equation as described previously^25^. Wsr = 2.13 × [(8 × 0.625 × Vcl)/Dv)], where Vcl is the center line velocity in mm/sec, Dv is the venule diameter in mm, and Wsr is in s^−1^. Vcl and Dv were calculated from measurements derived from infusion of FluoSpheres yellow-green fluorescent microspheres (0.2 μm, Molecular Probes) into the cremaster venule and analyzed using the SlideBook software. Wall shear stress (Wst) within the mouse cremaster venule was calculated using the following equation: Wst = Wsr × viscosity, where Wsr is in s^−1^ and viscosity is in poise [P]. A value of 0.035 [P] was used for the viscosity of blood.

### O_2_^•**−**^ Detection in Mice

Dihydroethidium (DHE) (Invitrogen, Grand Island, NY) was prepared as a 1 mg/ml solution in 1% Dimethyl sulfoxide (DMSO) and administered at 10 mg per kg of body weight by intravenous injection as described previously^29^,^55^. Mice were killed and immediately perfusion-fixed with 4% paraformaldehyde 1 hour after DHE injections. Frozen sections of ischemic muscles were prepared and observed by confocal microscope (Zeiss, Thornwood, NY) with excitation at 510–550 nm and emission >585 dsx7Anm to detect oxidized ethidium.

### In vivo measurement of luciferase gene expression by bioluminescence

HLL and Atox1^−/−^HLL mice were anesthetized, and the hair was removed over the back side of mice before imaging as described previously^56^. Luciferin (150 mg/kg/mouse in 200μl of isotonic saline) was administered by i.p. injection. After 12–16 min, mice were imaged with using Xenogen IVIS spectrum (Caliper Life Sciences, Hopkinton, MA, USA). For the duration of photon counting, mice were placed inside a light-tight box. Light emission from the mouse was detected as photon counts using the intensified charge-coupled device camera and expressed as photon counts. Images were obtained before and following hind-limb ischemic surgery. Luciferase activity was measured in ischemic tissue samples by adding 100 μl of freshly reconstituted luciferase assay buffer to 20μl of the homogenated ischemic tissue that was homogenized using reporter lysis buffer (Promega). Luciferase activity was expressed as relative light units normalized for protein content.

### Sponge implant model

To prepare sponge for implantation^22^, polyvinyl alcohol sponge (sponge CF120, 5mm diameter ×2.75 mm thick, Merocel) was hydrated using saline overnight and sterilized hydrated sponges by autoclaving for 25 min at 121 °C. Sterilized sponges were implanted into WT and Atox1 KO mice in the presence of VEGF. Implants were excised at 3 weeks following implantation for histological analysis because inflammatory angiogenesis peaks during this period as showed previously^22^. The sponge was harvested, fixed with paraformaldehyde and embedded in paraffin. The paraffin section (5 μM) was used for immunostaining and histology.

### Isolation of coronary vascular endothelial cells

Mouse coronary endothelial cells (MCECs) were isolated as previously described^57^. Initially, beads coated with sheep anti-rat IgG were incubated with purified rat anti-mouse CD31 monoclonal antibody (1μg/ml) at 4 °C overnight and then washed with PBS containing 0.1% BSA and 2 mM EDTA. Next day, dissected heart tissues were minced and incubated with M199 containing 1 mg/ml collagenase II for 1 h at 37 °C with mild agitation every 15 min. The digested material was filtered through sterile 40μm nylon mesh and washed in 2% fetal calf serum in M199. Cell suspensions were incubated with previously prepared antibody/beads complex for 30 min at 4 °C and beads/endothelial cells complex were isolated using magnet, washed and plated with medium containing endothelial growth factors.

### Quantitative real-time PCR

Total RNA of gastrocnemius and HUVECs was isolated by using phenol/chloroform and isolated using Tri Reagent (Molecular Research Center Inc.). Reverse transcription was carried out using high capacity cDNA reverse transcription kit (Applied biosystems) using 2 ug of total RNA. Quantitative PCR was performed with the ABI Prism 7000, the SYBR Green PCR kit (Qiagen) and the QuantiTect Primer Assay (Qiagen) for specific genes. Samples were all run in triplicates to reduce variability. Expression of genes was normalized and expressed as fold-changes relative to GAPDH.

### Cell culture and reagents

Human umbilical vein endothelial cells (HUVECs) were grown on 0.1% gelatin coating in EndoGRO basal medium (Millipore) containing 5% fetal bovine serum (FBS) as previously described^30^. Passage number did not exceed P7. Human epithelial kidney cells (HEK293) were used in promoter activity experiments. HEK293 were grown in 10% FBS DMEM media. Human TNFα (R&D Systems) was used in all *in vitro* experiments at 10ng/ml. Protein knockdown by siRNA (Ambion) in HUVECs was done by Oligofectamine (Invitrogen) for 3h and followed by 48h growth. Transfection of HEK293 by plasmid DNA was accomplished through Polyfect (Qiagen) according to manufacturer protocol. Transfection was allowed for 18h prior to treatment with harvest or TNFα treatment. THP-1 monocytes were grown in RPMI media containing 10% FBS.

### Monocyte Adhesion Assay

HUVECs were grown in 24-well glass bottom plates and treated for 18h with TNFα. THP-1 cells were labeled with Cell Tracker Green CMFDA (Invitrogen) and were added to the EC monolayer. Cultures were incubated in a CO_2_ incubator for 1h. Nonadherent cells were removed from the plate by gentle washing with PBS, and the number of adherent cells was determined by confocal microscopy. Cells were counted in five random 20× fields per well.

### H_2_O_2_ Measurement

H_2_O_2_ production was detected by incubating the cells with 20 μM 5-(and-6)-chloromethyl-2′,7′-dichlorodihydrofluorescein diacetate, acetyl ester (CM-H_2_DCFDA, Invitrogen) in the presence or absence of PEG-catalase for 6 min at 37 °C and observed by confocal microscopy using same exposure condition in each experiment. Relative DCF-DA fluorescence intensity was recorded and analyzed using LSM software as we reported previously^30^.

### Immunofluorescence analysis

HUVECs on glass coverslips were rinsed quickly in ice-cold PBS, fixed in freshly prepared 4% paraformaldehyde in PBS for 10 min at room temperature, permeabilized in 0.05% Triton X-100 in PBS for 5 min, and rinsed sequentially in PBS, 50 μmol/L NH_4_Cl and PBS for 10 min each. After incubation for 1 h in blocking buffer (PBS+3%BSA), cells were incubated with Atox1 antibody for 18 h at 4 °C, rinsed in PBS/BSA, and then incubated in Alexa Fluor 488-conjugated goat anti-rabbit IgG for 1 h at room temperature and cells rinsed with PBS. Cells on coverslips were mounted onto glass slides using Vectashield (Vector Laboratories) and observed using confocal microscopy.

### Immunoblotting

HUVECs were stimulated with TNFα for 18h and cells were lysed in lysis buffer, pH 7.4 (in mM) 50 HEPES, 5 EDTA, 120 NaCl), 1% Triton X-100, protease inhibitors (10 μg/ml aprotinin, 1 mmol/L phenylmethylsulfonyl fluoride, 10 μg/ml leupeptin) and phosphatase inhibitors (mmol/L) 50 sodium fluoride, 1 sodium orthovanadate, 10 sodium pyrophosphate). Cell lysates were used for immunoblotting, as described previously^30^. Goat anti-ICAM-1 (Santa Cruz), goat anti-VCAM-1 (Santa Cruz), and Mouse anti-actin (Santa Cruz) were incubated on nitrocellulose membranes containing protein.

For protein expression in ischemic muscle, mice were perfused with cold phosphate buffer saline. Muscle samples were harvested and frozen in liquid nitrogen. Muscle samples were crushed and lysed with RIPA lysis buffer (5 mM Tris-HCl (pH 7.6), 150 mM NaCl, 1% NP-40, 1%sodium deoxycholate, 0.1% SDS) with protease inhibitor followed by brief sonication as described previously^40^. Equal amount of protein was separated by SDS-PAGE. Following primary antibodies were used: anti-Atox1 (home-made), anti-p47phox (Santa Cruz), or anti-VEGF antibody (Abcam). Protein expression was visualized by ECL (Amersham). Band density was quantified by ImageJ.

### Capillary network formation assay

HUVECs transfected with control siRNA or Atox1 siRNA were seeded on top of the thick growth factor-reduced Matrigel-coated wells (BD Biosciences) and incubated for 6 h at 37 °C. Images were taken with a Nikon digital camera, and eight random fields per well were analyzed.

### Plasmids, Deletions, and Site-Directed Mutagenesis

The luciferase reporter vectors (pGL3-p47phox −1217/+52, −224/+52, −86/+52, and −36/+52) were generated as previously described^51^. Site mutagenesis (GAAAGA to TCCCTA) and deletion of the p47phox −1217/+52 construct (−110 to −105 region) was done using Quick-change II XL (Agilent Technologies). The Atox1 mutants for promoter activity studies (Flag-Atox1-WT, Flag-Atox1-K56, 60E, Flag-Atox1-C12, 15S) were generated as previously described^17^. Purified adenovirus expressing Atox1 (Ad-Atox1) or LacZ (Ad-LacZ) (control) were used for *in vivo* injection.

### Transient Transfection and Reporter Assay

HEK293 cells transfected with pGL3-p47phox constructs along with pRL-Null (Promega) according to the manufacturer’s protocol. One day after transfection, luciferase activity was assayed using a luminometer and normalized to *Renilla* luciferase activity produced by the co-transfected control plasmid pRL-Null. Samples were run in triplicate to reduce variability.

### Chromatin Immunoprecipitation (ChIP) Assay

HUVECs grown to confluence were treated with TNFα. ChIP assays were performed by following the EpiTect ChIP One-Day Kit protocol (SA Bioscience). Cells were treated with formaldehyde (final concentration of 1%) for 15 min at 37 °C to cross-link proteins to DNA before harvesting. Then cells were rinsed twice with ice-cold PBS containing protease inhibitor, scraped into conical tubes, and pelleted for 4 min at 2000 rpm at 4 °C. Cells were resuspended in 150μl of lysis buffer (1% SDS, 10mM EDTA, 50mM Tris, pH 8.1, containing protease inhibitor) and incubated for 10 min on ice. Resuspended cells were sonicated 10 times for 10s each on ice and a second round of 5 times for 5s each. After centrifugation, the supernatant was diluted 1:10 with dilution buffer (0.01% SDS, 1.1% Triton X-100, 1.2mM EDTA, 16.7 mM Tris-HCl, pH 8.1, 167 mM NaCl). The cell lysate was precleared by incubation at 4 °C for 1 h with 45μl of salmon sperm DNA/protein A-agarose beads. The cleared lysates were incubated with anti-p65/RelA (Santa Cruz), monoclonal Atox1 antibody (Z31112) as described above, histone (Santa Cruz), or normal mouse IgG overnight. The following primers were used to amplify ICAM-1: Forward 5′-TGTTCCCAGGTGAGTCGGGGTG-3′ and reverse 5′-TCGTGGAGGTATGCAGGGTCTGG-3′. VCAM-1: Forward 5′-AAATCAATTCACATGGCATA-3′ and reverse 5′- AAGGGTCTTGTTGCAGAGG-3′. -110 region of p47phox: Forward 5′-GCGACGGGAAGGACAAGTGTAAA-3′ and reverse 5′-GCTGCGCCTTAAATGCACTGGAAA-3′.

### LOX activity assay

LOX activity in cultured medium and ischemic tissue lysate was measured by a high-sensitivity fluorescence assay as previously described^35^. Protein samples was incubated in the presence and absence of 500 μmol/L BAPN at 37 °C for 30 min with final reaction mixture supplied by Amplite Fluorimetric Lysyl Oxidase Assay kit (AAT Bioquest) to the manufacturer’s instruction. The reaction was stopped on ice, and differences in fluorescence intensity (540-nm excitation wavelength and 590-nm emission wavelength) between samples with and without BAPN were determined. Specific activity was determined by the ratio of activity to relative amount of protein.

### Monoclonal Atox1 antibody production

Mouse monoclonal Atox1 antibody (Z3112) was developed by using baculovirus particles displaying the surface glycoprotein gp64-fusion protein as the immunizing agent. Briefly, the Atox1 cDNA was ligated into the gp64 gene to create a fusion protein that is expressed in the viral surface, as previously described^58^. Positive clones were selected using recombinant Atox1 protein or cells overexpressing Atox1 by adenovirus^17^,^32^.

### Statistical analysis

All the experiments were repeated at least three times, and all values were expressed as mean ± SE. Blood flow recovery in the ischemic hindlimb was compared between the two groups by two-way repeated measures ANOVA, followed by Bonferroni post hoc analysis. Comparison between groups was analyzed by unpaired Student 2-tailed t test (2 groups) or ANOVA for experiments with more than two subgroups followed by Bonferroni post hoc analysis. For clinical studies, the distributions of the data were scrutinised for normality using the Shapiro-Wilk test. Parametric and non-parametric pairwise comparisons were analysed with the Student’s *t*-test and Mann-Witney U test, respectively. Differences in proportions were tested using the *z*-test. All tests performed were 2-sided with a p < 0.05 considered statistically significant. Statistical tests were performed using Prism v6 (GraphPad Software, San Diego, CA).

## Additional Information

**How to cite this article**: Chen, G.-F. *et al.* Copper Transport Protein Antioxidant-1 Promotes Inflammatory Neovascularization via Chaperone and Transcription Factor Function. *Sci. Rep.* **5**, 14780; doi: 10.1038/srep14780 (2015).

## Supplementary Material

Supplementary Information

Supplementary Video 1

Supplementary Video 2

Supplementary Video 3

Supplementary Video 4
