# Supplementary material for: Copper Transport Protein Antioxidant-1 Promotes Inflammatory Neovascularization via Chaperone and Transcription Factor Function
Source: Sci Rep. 2015 Oct 6;5:14780. doi: 10.1038/srep14780 (PMC4594038; doi:10.1038/srep14780)
Supplement: Supplementary Information [file srep14780-s1.pdf]

SUPPLEMENTARY INFORMATION

Copper Transport Protein Antioxidant-1 Promotes Inflammatory Neovascularization via Chaperone and Transcription Factor Function

#Gin-Fu Chen[1], #Varadarajan Sudhahar [1,3,4], Seock-Won Youn[2,3], Archita Das[1,3], Jaehyung Cho[2], Tetsuro Kamiya[1,3], Norifumi Urao[2], Ronald D. McKinney [1,2,4], 'Bayasgalan Surenkhuu[1]; Takao Hamakubo[5], Hiroko Iwanari[5], Senlin Li[6], John W. Christman[7], Saran Shantikumar[8], Gianni D Angelini[8,9], Costanza Emanueli[8,9]
*Masuko Ushio-Fukai[2,3] and *Tohru Fukai[1,2,3,4]

From [1]Departments of Medicine (Section of Cardiology) and Pharmacology,
[2]Department of Pharmacology, [3]Center for Cardiovascular Research,
University of Illinois at Chicago, Chicago, IL,
[4]Jesse Brown Veterans Affairs Medical Center, Chicago IL,
[5]Department of Quantitative Biology and Medicine, Research Center for Advanced Science and Technology, University of Tokyo, Tokyo, Japan,
[6]Department of Medicine, University of Texas Health Science Center, San Antonio, Texas,
[7]Division of Pulmonary, Allergy, Critical Care and Sleep Medicine
The Ohio State University Wexner Medical Center, OH,
[8]Bristol Heart Institute, School of Clinical Sciences, University of Bristol, Bristol,
[9]National Heart and Lung Institute, Imperial College of London; London, UK

# and *, these authors equally contributed to this work.

Running title: *Atox1 and inflammatory neovascularization*

Address correspondence:

Tohru Fukai, M.D., Ph.D.
Depts. of Medicine and Pharmacology
University of Illinois at Chicago,
835 S. Wolcott, M/C868, E403 MSB
Chicago, IL60612
Tel: 312-996-7631
Fax: 312-996-1225
Email: tfukai@uic.edu

Masuko Ushio-Fukai, Ph.D.
Department of Pharmacology
University of Illinois at Chicago,
835 S. Wolcott, M/C868, E403 MSB
Chicago, IL60612
Tel: 312-996-7665
Fax: 312-996-1225
Email: mfukai@uic.edu

Supplementary Methods

Clinical Samples Analyses: Studies on clinical samples complied with the ethical principles stated in the Declarations of Helsinki and were covered by ethical approvals (NHS-NRES 11SW/0093 and 10/HO107/63, from the Central Bristol Research Ethics Committee). Anonymized limb muscle samples (adjacent to the surgical incision) were obtained from patients receiving limb amputation for critical limb ischemia (CLI) and from subjects with no peripheral vascular disease who were undergoing saphenous vein harvesting in preparation for elective coronary artery bypass grafting (non-CLI control). Patient characteristics are reported in the online-only Data Supplement (Supplement Table 1). All muscle samples were collected in RNALater® RNA stabilization solution (Life Technologies, Paisley, UK), incubated overnight at 4°C and stored at -80°C, as per the manufacturer's protocol.

RNA was isolated using Qiazol and the miRNA extraction kit miRNeasy (Qiagen, Crawley, UK). Approximately 50mg of tissue was added to 1ml of Qiazol in a MACS C tube (Miltenyi, Surrey, UK). Tissue was homogenised using the program RNA02 of the gentleMACS dissociator (Miltenyi). The manufacturer's protocol was then followed, with the RNA finally eluted in 100µl nuclease-free water. RNA concentration was quantified using the NanoDrop-1000 spectrophotometer (Nanodrop Instruments, Delaware, USA), and then stored at -80°C. For reverse transcription, cDNA was synthesised from 500 ng of total RNA using the QuantiTect RT kit (Qiagen), as per the manufacturer's instructions, and included a step to remove genomic DNA. All cDNA was stored at -20°C. Quantitative PCR was performed using Power SYBR (Life Technologies) with a LightCycler 480 (Roche, St Albans, UK), according to the manufacturer's instructions. Data were normalised to 18S ribosomal RNA as an endogenous housekeeping gene, and relative expression was calculated using the $2^{\wedge-(\Delta C_T)}$ method, where ΔC_T = test gene C_T – housekeeping gene C_T.

Supplementary Table 1

	**Control group (n = 11)**	**Critical limb ischemia group (n = 11)**	***p* value**
**Age**, years (median and IQR)	67 (64 to 78)	66 (59 to 79)	0.49
**Male**, n (%)	9 (82%)	9 (82%)	1.00
**Hypertension**, n (%)	10 (91%)	8 (73%)	0.27
**Diabetes**, n (%)	3 (27%)	11 (100%)	< 0.01
**Insulin therapy**, n (%)	1 (9%)	4 (36%)	0.13
**Coronary Arterial Disease**, n (%)	11 (100%)	8 (73%)	0.06
**Creatinine**, mg/dl (mean ± SEM)	1.12 ± 0.07	1.17 ± 0.16	0.79
**Chronic Renal Failure**, n (%)	3 (27%)	3 (27%)	1.00
**Statins**, n (%)	8 (73%)	4 (36%)	0.09
**Aspirin**, n (%)	10 (91%)	3 (27%)	< 0.01
**Clopidogrel**, n (%)	4 (36%)	5 (45%)	0.67

Supplementary Table 1. Clinical characteristics of patients with or without critical limb ischemia. Leftover limb muscle samples from patients undergoing cardiovascular surgery were analysed. IQR = interquartile range, SEM = standard error of the mean. *p* values were calculated using Mann-Witney test (for age), unpaired *t*-test (for creatinine) or the z-test for proportions (for all other variables).

Supplementary Figures

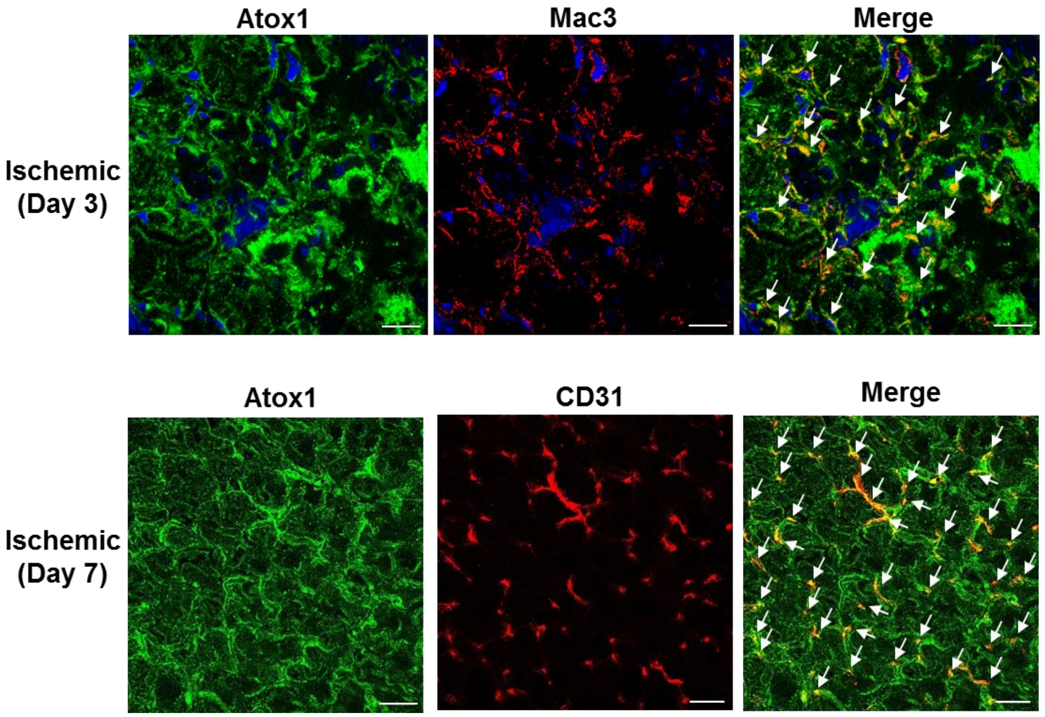

Supplementary Figure 1. Atox1 is expressed in CD31^{+} endothelial cells and Mac3+ macrophages in ischemic hindlimbs. Representative staining for Atox1 (green), Mac3 (macrophage marker, red), or their colocalization (merge) at day 3, and Atox1 (green), CD31 (EC marker, red), or their colocalization (merge) at day 7 after ischemia in gastrocnemius muscles. White arrows represent colocalization with Atox1/Mac3 (upper) or Atox1/CD31 (lower). Bars represent 100um.

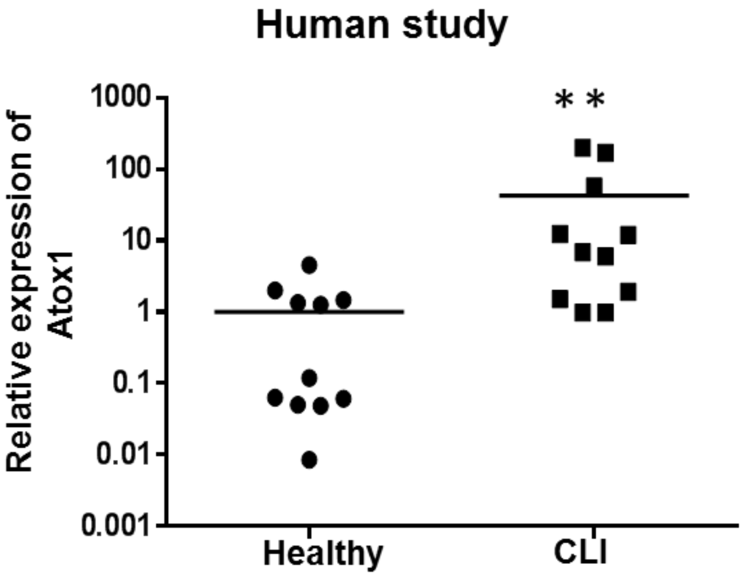

Supplementary Figure 2. Atox1 is increased in ischemic muscles from critical limb ischemia patients. Atox1 mRNA expression in limb muscles of patients with and without CLI. Expression is given relative to 18S (n=11).

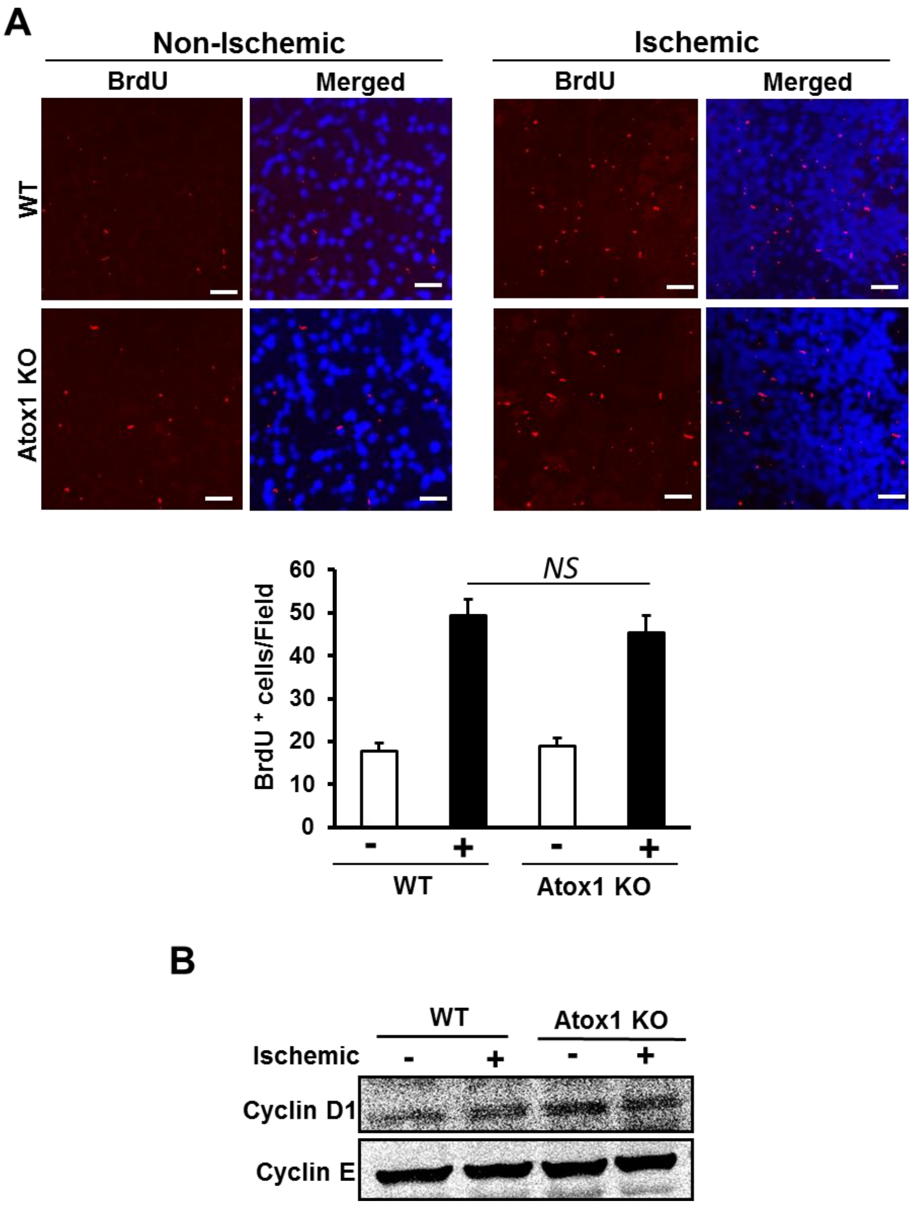

Supplementary Figure 3. **Atox1 is not involved in cell proliferation in ischemic hindlimbs. A and B**, Representative staining of BrdU (red) and DAPI (blue) (A) and western analysis for cyclin D1 protein expression (B) in non-ischemic and ischemic gastrocnemius muscles at 5 days after hindlimb ischemia. In A, scale bars= 20μm.

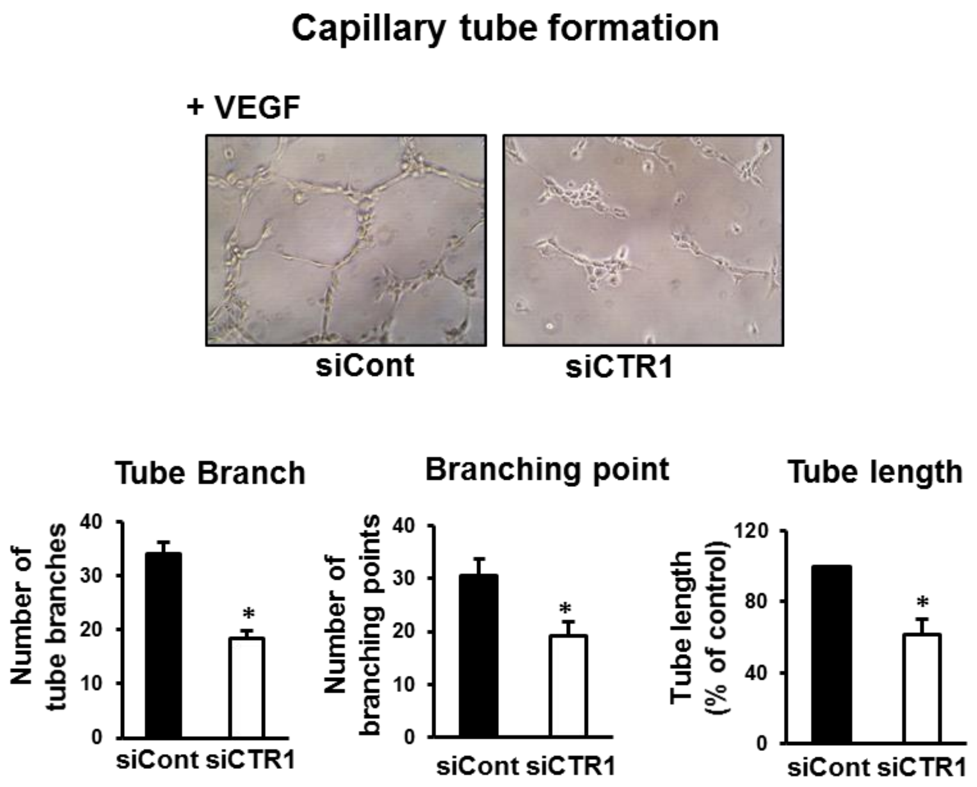

Supplementary Figure 4. Cu importer CTR1 is required for VEGF-induced capillary tube formation. HUVECs were transfected with control or CTR1 siRNAs and seeded on Matrigel-coated plates in culture media containing VEGF for 6 h. Four random fields per well were imaged, and representative pictures are shown (Top). Averaged numbers of capillary tube branches, branching points, and tube length per field are shown (Bottom). * $p < 0.05$.

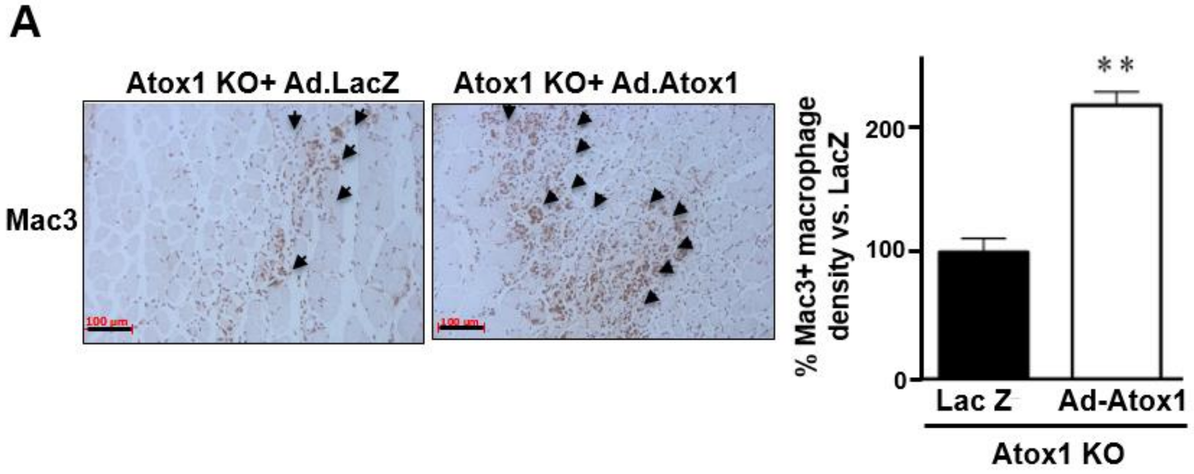

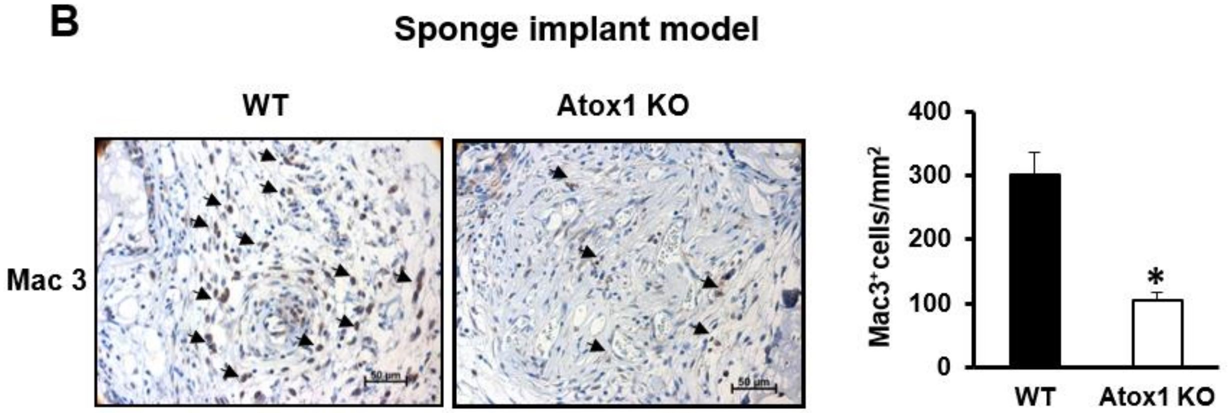

Supplementary Figure 5. Atox1 is required for inflammatory cell recruitment to the ischemic sites *in vivo* A, Representative images of Mac3 staining in ischemic limbs at day 3 in Atox1 KO mice injected with purified recombinant adenoviruses (Ad.Atox1-WT, Ad. LacZ (control), $1x10^9$ pfu) into the adductor and gastrocnemius muscles at one day prior to surgery. Graphs are summary of $Mac3^+$ cells at 2-3 ischemic regions per section (n=4, ** p<0.01 vs. WT. Scale Bars=100 µm. **B,** Polyvinyl alcohol sponge was implanted subcutaneously into WT and Atox1 KO mice. Representative images for immunostaining for macrophage infiltration (Mac3) in sponges harvested on day 21. Lower panels show quantitative analysis of the number of Mac 3^+ cells (n=5,* p <0.05). Scale Bars= 50µm.

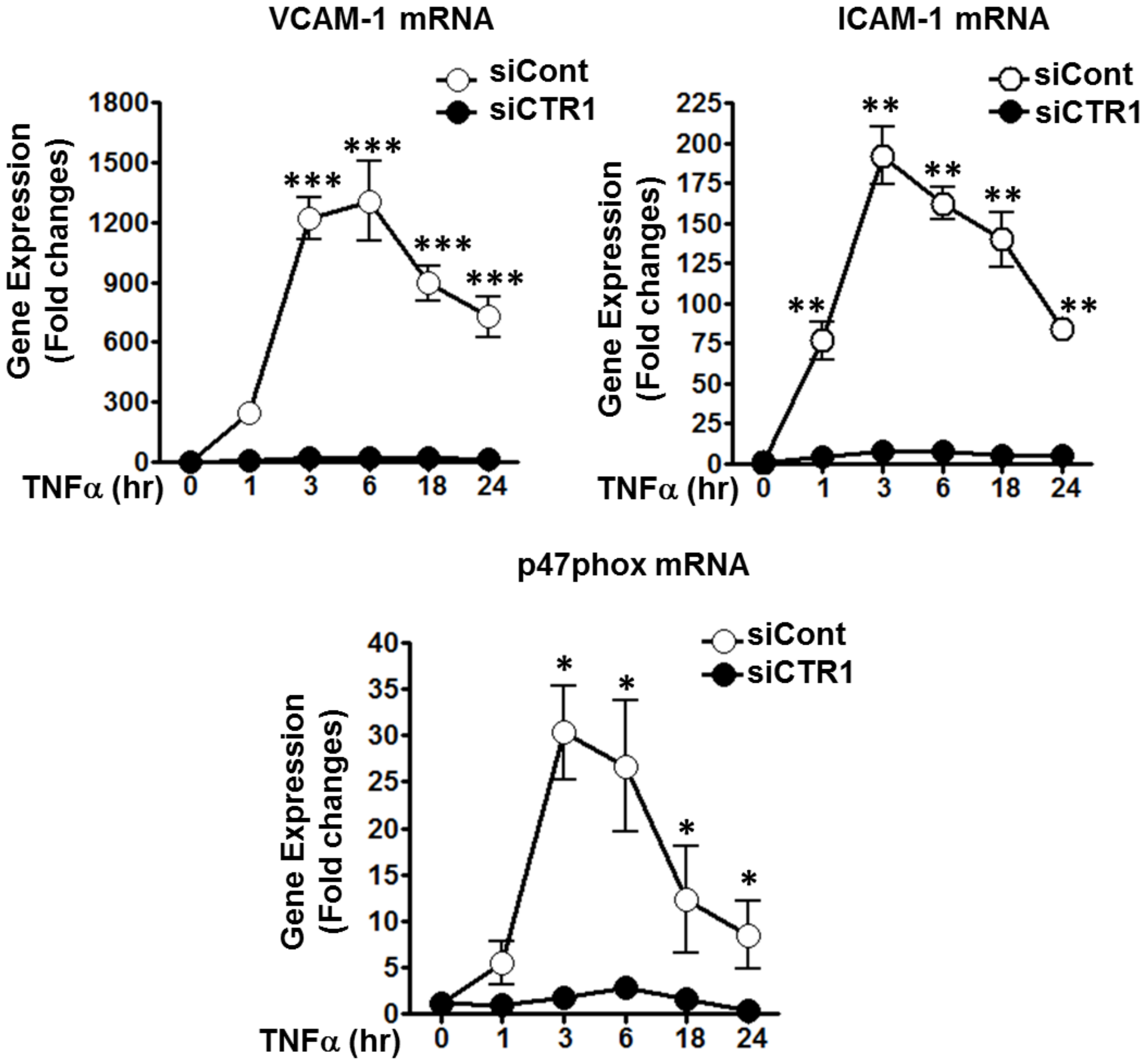

Supplementary Figure 6. Cu importer CTR1 is required for TNFα-induced expression of adhesion molecules and p47phox in ECs. HUVECs were transfected with CTR1 or control siRNAs for 48 hours. Cells were then incubated with TNFα (10ng/ml) for the time indicated. The mRNA levels of each gene were assessed by quantitative real-time PCR. Gene analysis is taken from 4 samples per group taken from 2 independent experiments with samples run in triplicate. *p<0.05, ** p<0.01 and *** p<0.001 vs. untreated control cells.

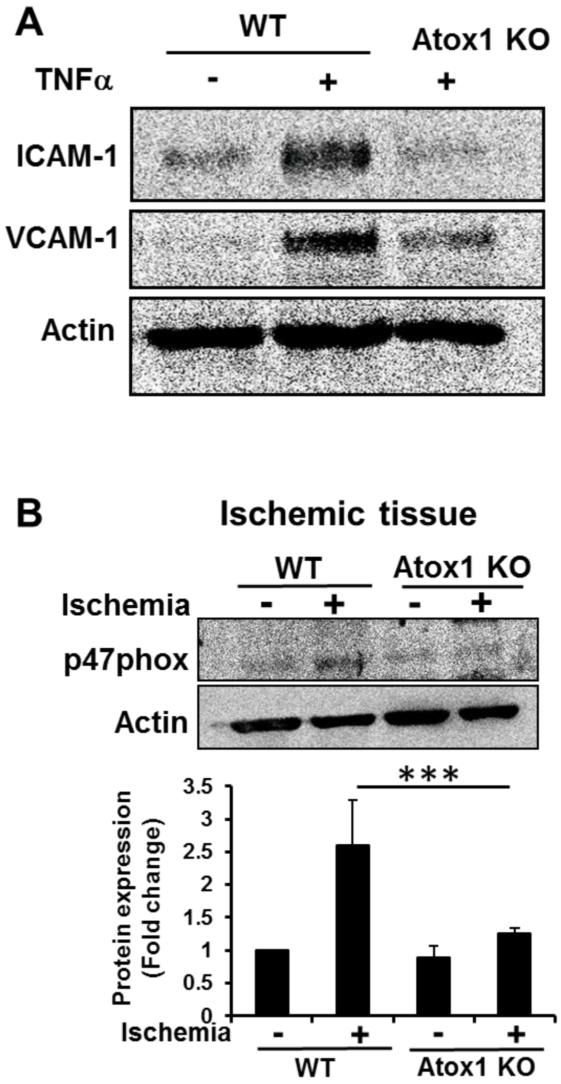

Supplementary Figure 7. Reduced ICAM-1/VCAM-1 expression in Atox1 KO ECs activated by TNFα and reduced p47phox protein expression in Atox1 KO ischemic tissue. A, ECs isolated from WT and Atox1 KO mice stimulated with TNFα for 18 hours were used to measure VCAM, ICAM-1 and actin protein expression. Representative blots from 3 different experiments. **B**, Ischemic and non-ischemic tissues of WT and Atox1 KO mice at day 7 were used to measure p47phox and actin protein expression. (n=3). ***p<0.001 vs ischemic tissue.

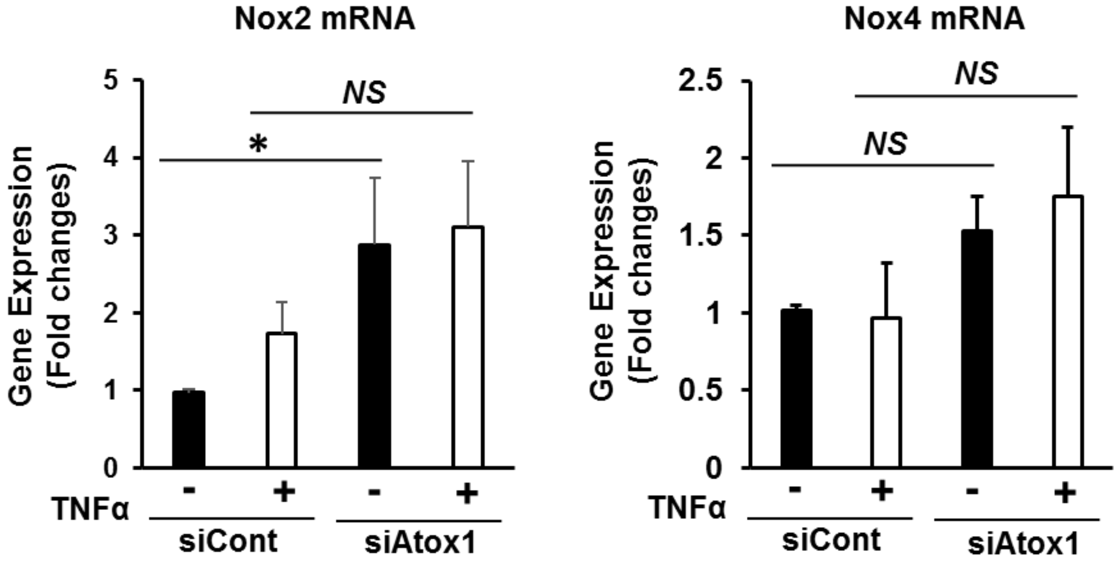

Supplementary Figure 8. Atox1 depletion with siRNA does not reduce Nox2 and Nox4 mRNA expression in TNFα-treated ECs. HUVECs were transfected with Atox1 or control siRNAs for 48 hours. Cells were then incubated with TNFα (10ng/ml) for the 3 hrs. The mRNA levels of each gene were assessed by quantitative real-time PCR. Gene analysis is taken from 3 independent experiments.

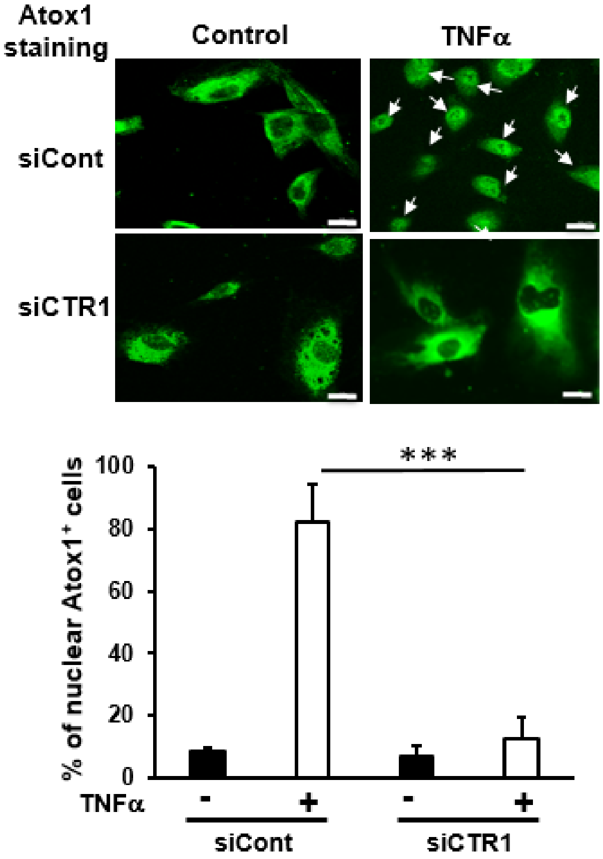

Supplementary Figure 9. Cu importer CTR1 is required for TNFα-induced Atox1 nuclear translocation in ECs. Immunofluorescence staining of Atox1. HUVECs were transfected with Ctr1 or control siRNAs and stimulated with TNFα (10 ng/ml) for 30 min. Cells were immunostained with anti-Atox1 antibody. Scale bars= 20μm. Percentage of Atox1+ cells in nucleus was shown in bottom (n=3).

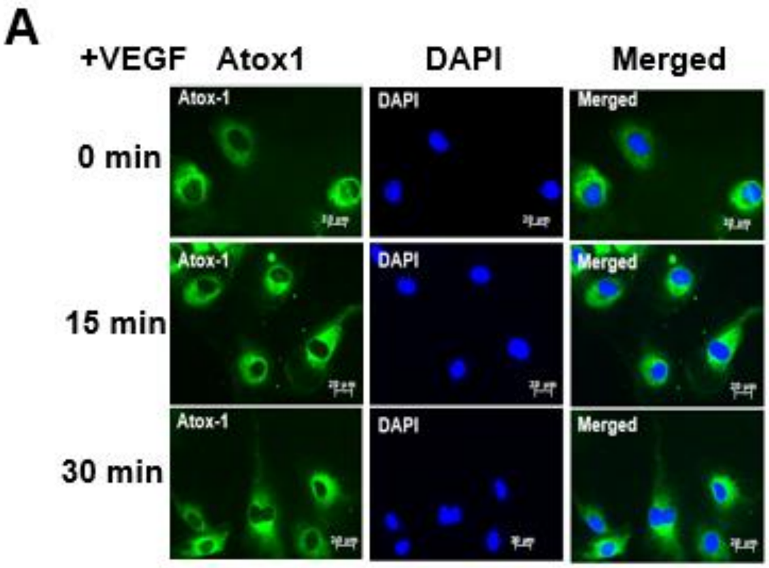

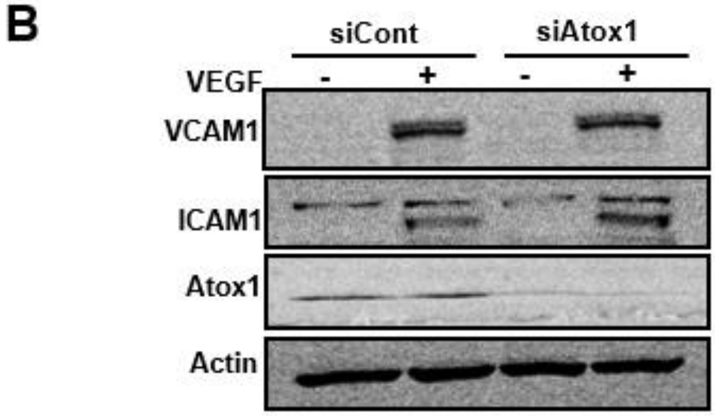

Supplemental Figure 10. A, VEGF does not promote Atox1 translocation to the nucleus in ECs. HUVECs were stimulated with VEGF (20 ng/ml) for indicated time and cells were stained with anti-Atox 1 antibody and the nuclear marker, DAPI. In each image, percentage of nuclear Atox1 positive cells was calculated from 5 randomized view and the cell images are representative of 3 different experiments. **B, Atox1 depletion by siRNA has no effects on VEGF-induced ICAM1/VCAM1 expression in ECs.** Cells transfected with Atox1 or control siRNAs were incubated with VEGF (20 ng/ml) for 4 hrs, and VCAM-1 and ICAM-1 protein expression was measured by Western analysis. Actin was used for a loading control

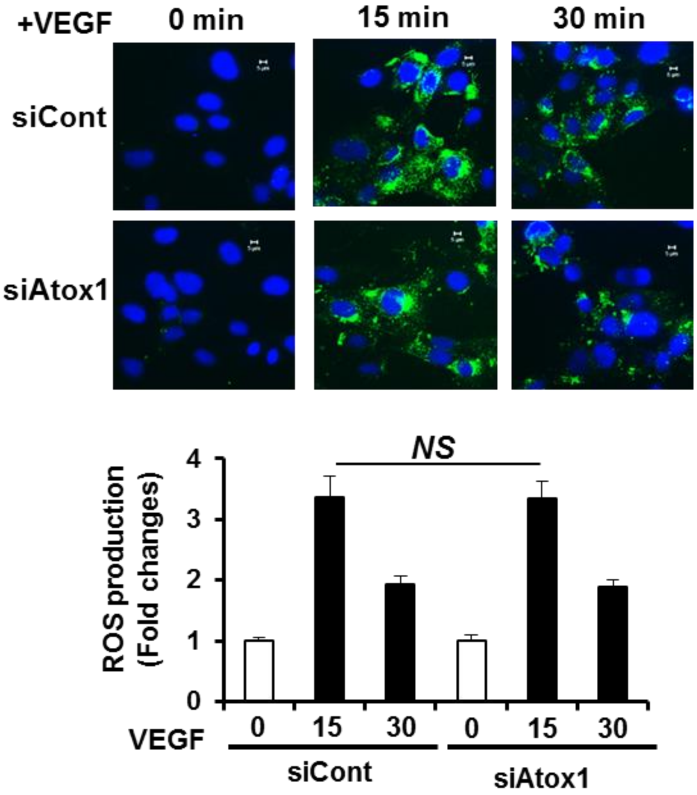

Supplementary Figure 11. Atox1 depletion by siRNA has no effects on VEGF-induced ROS production in ECs. HUVECs transfected with Atox1 or control siRNAs were stimulated with VEGF (20ng/ml) for the time indicated. Representative images for DCF fluorescence and DAPI staining (blue, nucleus marker) (top) and quantification of fluorescence intensity (bottom) (n=3) are shown.

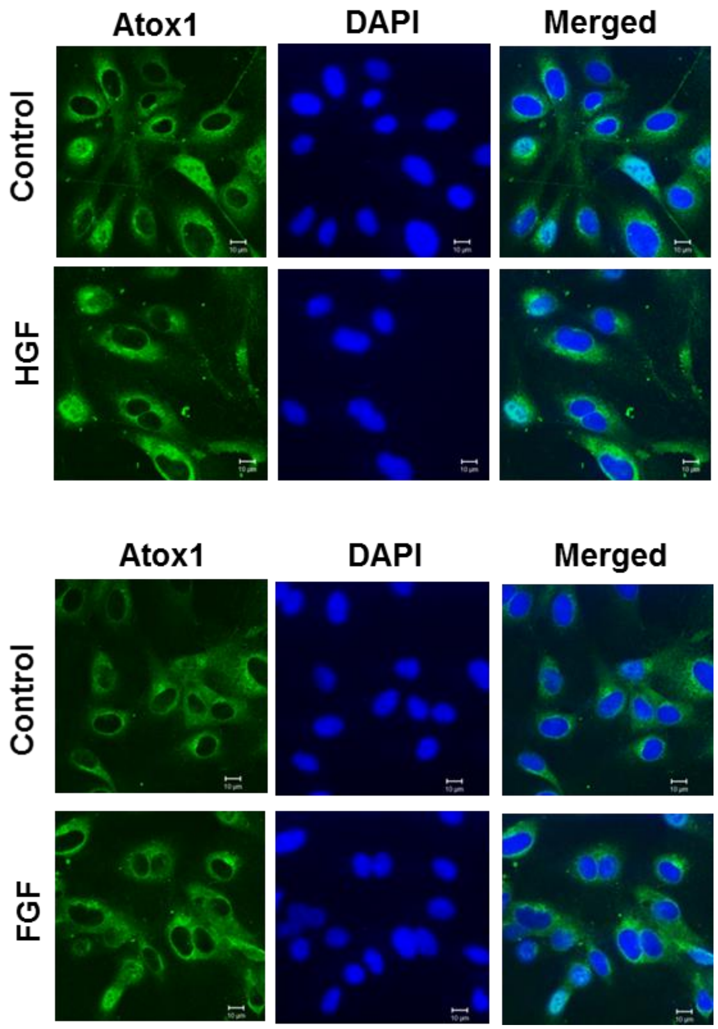

Supplementary Figure 12. Hepatocyte growth factor (HGF) and Fibroblast growth factor (FGF) do not promote Atox1 translocation to the nucleus in ECs. HUVECs were stimulated with HGF (20 ng/ml) or FGF (10 ng/ml) for 30 min. Cells were immunostained with anti-Atox1 antibody and the nuclear marker DAP1. Scale bars= 20μm.

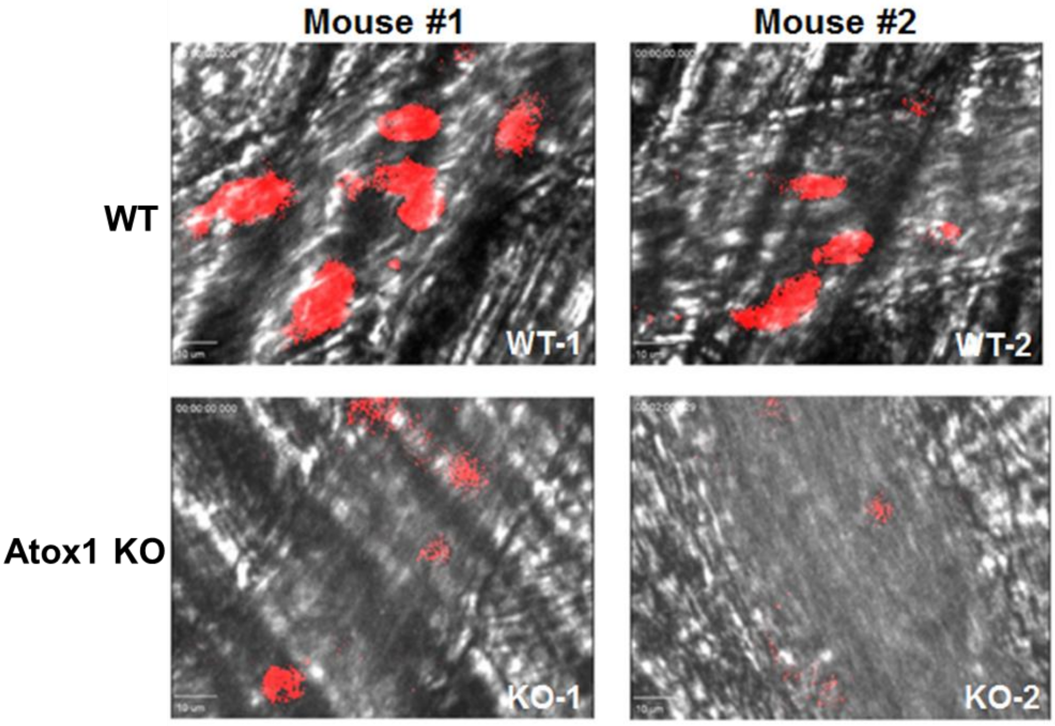

Supplementary Video 1. Real-time intravital microscopic analysis of neutrophil rolling and adhesion on the TNFα-inflamed endothelium of postcapillary venules of cremaster muscle in WT and Atox1 KO mice. TNFα was intrascrotally injected into WT and Atox1 KO mice. At three hours after TNFα injection, the cremaster muscle was exposed. Mouse neutrophils were monitored in an area of 0.02 mm^2 over 5 min in the inflamed cremaster muscle venules by infusion of Alexa 647-labeled anti-Gr-1. 10 sec time lapse are shown. A video of two different mice from each group (i.e. WT vs. Atox1 KO) are shown (S1video-WT-1, S1video-WT-2, S1video-KO-1, S1video-KO-2).
